# Alanine and glutathione targeting of dopamine- or ibuprofen-coupled polypeptide nanocarriers increases both crossing and protective effects on a blood–brain barrier model

**DOI:** 10.1186/s12987-025-00623-2

**Published:** 2025-02-19

**Authors:** Mária Mészáros, Thi Ha My Phan, Judit P. Vigh, Gergő Porkoláb, Anna Kocsis, Anikó Szecskó, Emese K. Páli, Nárcisz M. Cser, Tamás F. Polgár, Gábor Kecskeméti, Fruzsina R. Walter, Jens C. Schwamborn, Tamás Janáky, Jeng-Shiung Jan, Szilvia Veszelka, Mária A. Deli

**Affiliations:** 1 Institute of Biophysics, HUN-REN Biological Research Centre, Temesvári Krt. 62, 6726 Szeged, Hungary; 2 Faculty of Health Sciences, One Health Institute, University of Debrecen, Nagyerdei Krt. 98, 4032 Debrecen, Hungary; 3 Department of Chemical Engineering, National Cheng Kung University, Tainan, 70101 Taiwan; 4 Doctoral School of Biology, University of Szeged, Dugonics Tér 13, 6720 Szeged, Hungary; 5 Theoretical Medicine Doctoral School, University of Szeged, Tisza Lajos Krt. 97, 6722 Szeged, Hungary; 6 Department of Medical Chemistry, Albert Szent-Györgyi Medical School, University of Szeged, Dóm Tér 8, 6720 Szeged, Hungary; 7 Luxembourg Centre for Systems Biomedicine (LCSB), Developmental and Cellular Biology, University of Luxembourg, 4365 Belvaux, Luxembourg; 8 Present Address: Smurfit Institute of Genetics, Trinity College Dublin, Dublin, Ireland

**Keywords:** Blood–brain barrier, In vitro model, Human stem cell derived endothelial cell, Poly(l-glutamic acid), Dual-targeted nanocarriers, Alanine, Glutathione, Dopamine, Ibuprofen

## Abstract

**Background:**

Targeting the blood–brain barrier (BBB) is a key step for effective brain delivery of nanocarriers. We have previously discovered that combinations of BBB nutrient transporter ligands alanine and glutathione (A-GSH), increase the permeability of vesicular and polypeptide nanocarriers containing model cargo across the BBB. Our aim was to investigate dopamine- and ibuprofen-coupled 3-armed poly(l-glutamic acid) nanocarriers targeted by A-GSH for transfer across a novel human co-culture model with induced BBB properties. In addition, the protective effect of ibuprofen containing nanoparticles on cytokine-induced barrier damage was also measured.

**Method:**

Drug-coupled nanocarriers were synthetized and characterized by dynamic light scattering and transmission electron microscopy. Cellular effects, uptake, and permeability of the nanoparticles were investigated on a human stem cell-based co-culture BBB model with improved barrier properties induced by a small molecular cocktail. The model was characterized by immunocytochemistry and permeability for marker molecules. Nanocarrier uptake in human brain endothelial cells and midbrain organoids was quantified by spectrofluorometry and visualized by confocal microscopy. The mechanisms of cellular uptake were explored by addition of free targeting ligands, endocytic and metabolic inhibitors, co-localization of nanocarriers with intracellular organs, and surface charge modification of cells. The protective effect of ibuprofen-coupled nanocarriers was investigated against cytokine-induced barrier damage by impedance and permeability measurements.

**Results:**

Targeted nanoformulations of both drugs showed elevated cellular uptake in a time-dependent, active manner via endocytic mechanisms. Addition of free ligands inhibited the cellular internalization of targeted nanocarriers suggesting the crucial role of ligands in the uptake process. A higher permeability across the BBB model was measured for targeted nanocarriers. After crossing the BBB, targeted dopamine nanocarriers subsequently entered midbrain-like organoids derived from healthy and Parkinson’s disease patient-specific stem cells. The ibuprofen-coupled targeted nanocarriers showed protective effects against cytokine-induced barrier damage.

**Conclusion:**

BBB-targeted polypeptide nanoparticles coupled to therapeutic molecules were effectively taken up by brain organoids or showing a BBB protective effect indicating potential applications in nervous system pathologies.

**Graphical Abstract:**

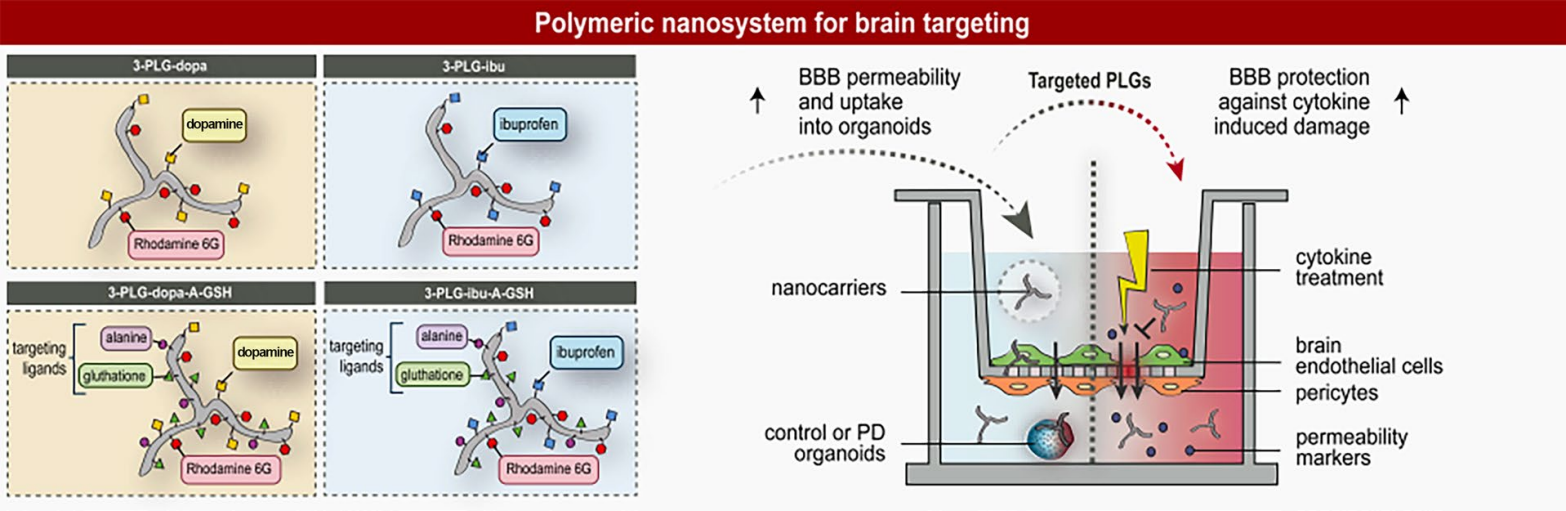

**Supplementary Information:**

The online version contains supplementary material available at 10.1186/s12987-025-00623-2.

## Introduction

The tight junction-connected, non-fenestrated brain capillary endothelial cells in cross-talk with the neighboring pericytes and astroglial endfeet create the dynamic anatomical and functional unit called as blood–brain barrier (BBB) [1]. The BBB highly controls the neuronal microenvironment by supplying the brain with nutrients and protecting it from potentially harmful molecules [2]. Most newly developed pharmacon candidates to treat neurological diseases are not able to cross the BBB and reach their target(s) within the brain [3]. Therefore, the effective therapy of many neurodegenerative disorders of the central nervous system (CNS) such as Parkinson’s disease (PD) or Alzheimer’s disease (AD) remains a serious challenge.

The brain tissue of PD patients is typically characterized by the death of dopaminergic, neuromelanin-containing neurons in the area of locus coeruleus and substantia nigra. The significant cell damage and loss result in decreased dopamine concentration within the striatum and the dysfunction of the nigrostriatal pathway. These changes cause serious motor symptoms in PD patients [4]. Dopamine supplementation would be evident, but this hydrophilic neurotransmitter does not cross the BBB and cannot enter the CNS due to brain endothelial tight junctions, active brain endothelial metabolism and lack of transporters [5]. Only the chemically modified levo-3,4-dihydroxy-phenylalanine (levodopa; l-dopa) can cross the BBB and reach brain tissue via l-type amino acid transporter-1 (LAT-1), the member of solute carrier (SLC) family present at the brain endothelium and diverse brain cells [6]. Despite the pharmacokinetic limitations, irregular adsorption and fast catabolism, the l-dopa became the standard oral pharmaceutical therapy of PD in the last fifty years [7].

Inflammation and dysfunction of the BBB play central role in the pathomechanisms of both PD and AD [8–10]. Protection of the BBB, the inhibition of the elements of the brain microvascular damage such as impairment of tight junctions, increased vesicular transcytosis, production of various cytokines, chemokines and reactive oxygen species offers novel therapeutic targets [11]. AD is characterized by two core pathologies, formation of amyloid-β plaques and neurofibrillary tangles. Inflammation in the CNS increases the risk of AD initiation and exacerbates the amyloid and tau pathology and the cognitive decline [12]. Several studies confirm that long-term treatment with non-steroidal anti-inflammatory drugs (NSAIDs) that non-selectively inhibit cyclooxygenases COX-1 and COX-2, such as ibuprofen, reduce the risk of AD by the inhibition of the inflammatory cascade [13–15]. NSAIDs can delay disease onset, ameliorate symptomatic severity, and slow cognitive decline [15]. The physicochemical characteristics of ibuprofen hinder its brain delivery. Ibuprofen is a lipophilic and neutral molecule but it becomes fully ionized in the systemic circulation at the pH of blood. This change makes the molecule too hydrophilic to cross BBB, therefore high dose is needed for CNS application [16]. The serious gastrointestinal side effects of long-term administration of NSAIDs including ibuprofen, limit their clinical applicability [17].

Nanosized carriers are promising drug delivery systems for the brain [18, 19]. The requirements of potential nanocarriers for CNS drug delivery are biodegradability, non-toxic characteristics, scalable synthesis and controlled loading or coupling with hydrophilic or hydrophobic drugs [20, 21]. Polypeptide-based nanocarriers, especially the l-isoform of poly(glutamate) conjugates, meet most of the expectations [22, 23]. The relatively easy ring opening polymerization of α-amino-*N*-carboxyanhydrides provides a wide variety of polypeptide structures such as homopolymers, copolymers, block copolymers, and multibranched nanocarriers [22]. Despite their good properties and high drug-coupling capacity poly(l-glutamic acid) nanosystems have been rarely investigated for brain drug delivery [23, 24].

To enable nanoparticles to cross the BBB and reach the CNS specific targeting is needed [21, 25]. The use of targeted nanocarriers solves the problems of both insufficient brain penetration of hydrophilic therapeutic molecules and high treatment doses inducing side-effects at the periphery [21, 26]. Influx transport systems at the BBB include SLCs and receptor-mediated endocytosis that contribute to nutrient delivery [2, 27]. The receptor-mediated transport systems are the most investigated for targeting nanoparticles, the surface of the majority of nanosystems is coupled with BBB receptor ligands. Based on the literature, ligands of the transferrin, insulin and leptin receptors, and low density lipoprotein receptor-related proteins (LRPs) are used to shuttle pharmaceutical agents across the BBB in preclinical studies [18, 28]. Adsorptive-mediated transport at the BBB has also been exploited to target nanocarrier systems by using cationic lipids and cell penetrating peptides to increase binding and internalization at the brain capillary endothelium [29, 30], which has a highly negative surface charge [31]. SLCs has become a prominent strategy for brain drug delivery of nanoparticles by functionalization with ligands such as hexoses, amino acids or vitamins [18]. The effectivity of BBB-specific cellular internalization and permeability can be increased by the combination of different targeting ligands on the surface of nanocarriers. Our research group investigated several ligands of BBB nutrient transport systems and the combination of the amino acid alanine and among them the tripeptide glutathione (A-GSH) was the most successful for targeting the BBB in case of nanovesicles [32, 33] or 3-armed poly(l-glutamic acid) (3-PLG) nanocarriers in BBB cultures and animals [24]. However, in our previous experiments fluorescent model compounds were loaded or coupled as cargo molecules and therapeutic agents were not investigated yet to prove the concept.

A major point in benchmarking targeted nanosystems is the prediction of in vivo human brain penetration. To study the permeability of nanoparticles in vitro BBB models, which conform to the “3R” principles with the minimalization the number of animal experiments, are widely used either in static [34] or in dynamic conditions by employing lab-on-a-chip systems [35]. To avoid problems due to species specific differences in drug transporters at the BBB [36] human cell-based models are preferable. Human induced pluripotent [37, 38] or hematopoietic [39, 40] stem cell-based models overcome the translational problems related to the BBB. One of the limitations of BBB models prepared from stem cell derived vascular endothelial cells is to induce appropriate barrier tightness and BBB characteristics. Our group has recently invented a molecular combination (cARLA) synergistically targeting signaling pathways related to BBB maturation that elevated the barrier tightness and other BBB-specific properties in several BBB culture models including human stem cell-based ones [40]. We also demonstrated that cARLA treatment improved the predictive value of a human stem cell derived co-culture BBB model not only for small molecule drugs but also for targeted nanoparticles [40].

The aim of this study was to prove our hypothesis that A-GSH functionalization increases brain endothelial internalization and BBB permeability of polymeric nanoparticles coupled with active agents, namely dopamine and ibuprofen, which have low brain penetration. As an experimental system we used a human BBB co-culture model improved by cARLA. To better understand the cellular uptake mechanisms of targeted nanocarriers we investigated the effects of free ligands, metabolic and endocytic inhibitors, and the modification of brain endothelial surface charge. The entry of dopamine-coupled nanocarriers into midbrain organoids derived from healthy or PD patients was also studied. Finally, we explored the protective effect of ibuprofen-coupled nanocarriers against cytokine-induced BBB damage.

## Materials and methods

### Materials

All reagents were purchased from Merck Life Science Ltd., Hungary, unless otherwise indicated. For the polypeptide synthesis all the chemicals were used directly as the products received from the vendors. Calcium hydride was used to dehydrate hexane and dichloromethane (DCM) while sodium and benzophenone were used as a dehydrator and a color indicator, respectively, for the water removal of tetrahydrofuran (THF). All the reactions were operated under a nitrogen atmosphere using a Schlenk-line system and a glovebox to protect air- and moisture-sensitive chemicals.

### Cell culture and characterization

The human BBB co-culture model based on stem cell-derived endothelial cells (hEC) and brain pericytes (PC) were established and characterized by the group of Ceccelli et al. [39]. This model was used by our team for nanoparticle studies [24], and its BBB properties were further enhanced by targeting signaling pathways with cARLA [40]. Isolation of CD34^+^ hematopoietic stem cells from human umbilical cord blood, differentiation towards the endothelial lineage, and to brain-like endothelial cells by co-culture with PCs were previously described in detail [39]. Endothelial cells (P6) were cultured in collagen type IV (100 µg/ml in distilled water; DW) and fibronectin (25 µg/ml in DW) coated dishes and in ECM-NG culture medium (Sciencell, USA) supplemented with 5% fetal bovine serum (FBS; Sciencell), 1% endothelial growth supplement (Sciencell) and 50 μg/ml gentamicin. In those experiments where using contact co-culture model was not possible, hECs received 50% conditioned medium from PCs to promote brain-like properties. PCs (≤ P11) were seeded into collagen type IV and fibronectin coated dishes in Dulbecco’s modified Eagle’s medium (DMEM, Life Technologies, Thermo Fisher Scientific, USA) supplemented with 20% FBS, 1% Glutamax (Life Technologies) and gentamicin (50 μg/ml). Conditioned media was collected from the PC cultures in the second day after the seedings and mixed in a 1:1 ratio with the medium of hEC [24].

To further enhance the BBB properties of the models, the medium was supplemented with cARLA (250 µM 8-(4-chlorophenylthio)adenosine 3′,5′-cyclic monophosphate sodium salt, 17.5 μM Ro-20-1724, 3 mM LiCl, 3 μM A83-01; 48 h) as described previously [40]. For characterization of cARLA-treated hECs the cells were cultured in 50% PC-conditioned medium on glass bottom chamber slides (Nunc™ Lab-Tek™, Thermo Fisher Scientific) coated with collagen IV and fibronectin at a seeding density of 5 × 10^4^ cells/cm^2^. After 48 h incubation of confluent monolayers with cARLA, the culture medium was removed and hECs were fixed with a 1:1 mixture of ice cold methanol-acetone solution for 2 min. Cells were kept in phosphate-buffered saline (PBS; KCl 2.7 mM, KH_2_PO_4_ 1.5 mM, NaCl 136 mM, Na_2_HPO_4_ × 2 H_2_O 6.5 mM, pH 7.4) containing 3% bovine serum albumin (BSA-PBS) for 1 h at room temperature and were then incubated with a rabbit anti-claudin 5 primary antibody diluted in 3% BSA-PBS (1:300) overnight at 4 °C. These steps were followed by incubation with a goat anti-rabbit IgG secondary antibody labeled with Alexa fluor 488 (1:400; Thermo Fisher Scientific) and the nuclear stain Hoechst 33342 (1 μg/ml; Thermo Fisher Scientific) diluted in PBS for 1 h at room temperature under protection from light. Between each step, cells were washed three times in PBS. Slides were mounted with Fluoromount-G (Southern Biotech, USA) and examined using a Leica TCS SP5 AOBS confocal laser scanning microscope (Leica Microsystems, Germany) equipped with HCX PL APO 63 × oil (NA = 1.4) objectives.

Expression of genes encoding alanine transporters in the cARLA-treated human co-culture model of BBB are shown from our previously published dataset publicly available at the Gene Expression Omnibus (GEO) under accession number GSE224846 [40]. Transcript per million values were shown from cARLA-treated hECs (samples GSM7034228, GSM7034229, GSM7034230 and GSM7034231).

The establishment, maintenance and characterization of midbrain organoids from human floor plate neuronal progenitor cells derived from a healthy (ID number: #232) and a Parkinson’s disease patient with triplication in the SNCA gene (ID number: #317) were previously described by Muwanigwa et al. [41]. These midbrain organoids were used in our groups previous studies with different types of nanoparticles [24, 42].

### Nanocarrier synthesis

#### Synthesis of 3-armed poly(γ-benzyl-l-glutamic acid) (3-PBLG)

**Fig. 1:**
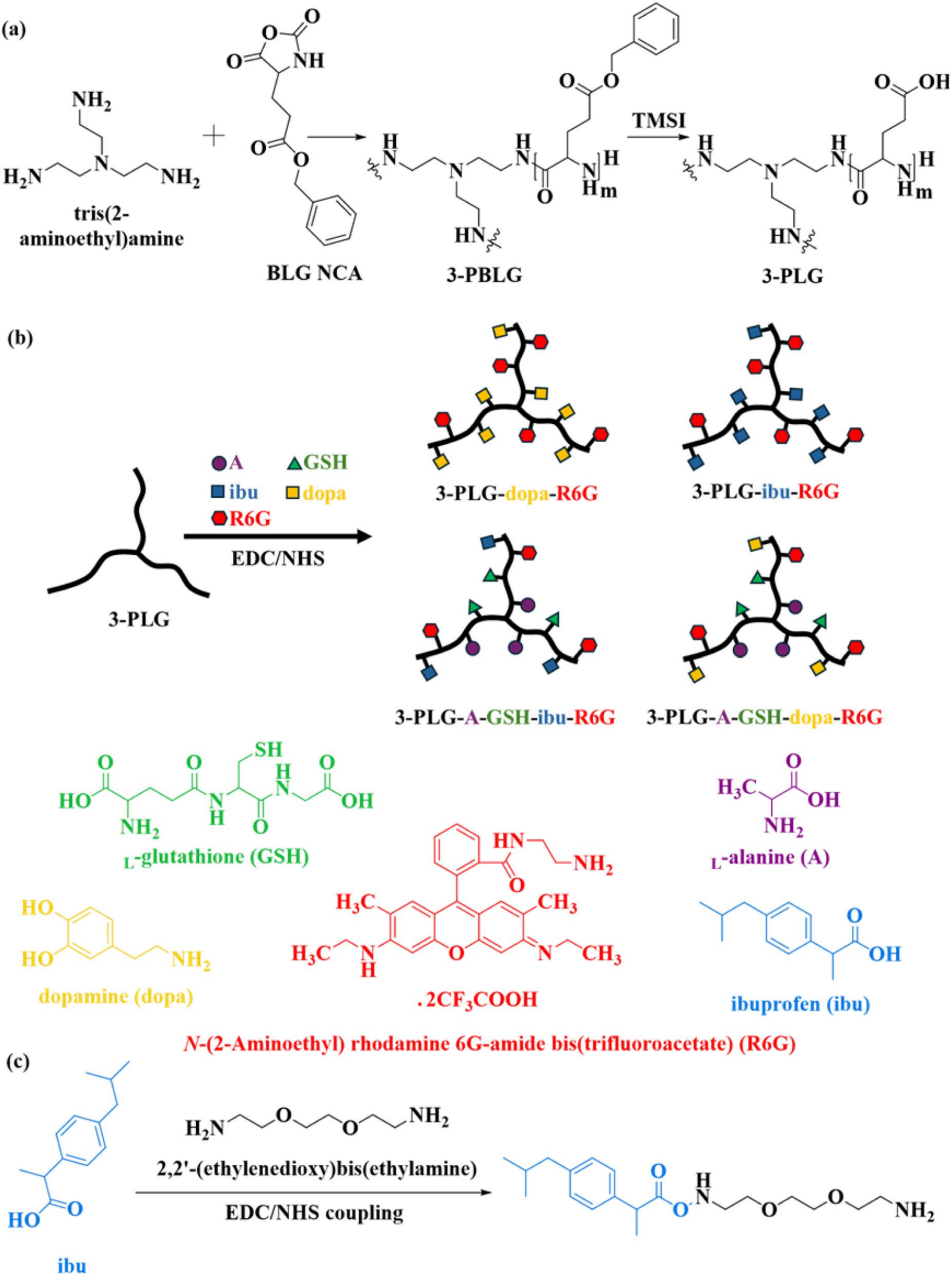
Synthesis of nanocarriers. **a** Synthesis and debenzylation of poly(γ-benzyl-l-glutamic acid) 3-PBLG. **b** Synthesis of copolypeptides using EDC/NHS coupling, and **c** modification of ibuprofen (ibu)

In this study, tris(2-aminoethyl)amine was used as a 3-armed initiator for the ring-opening polymerization method to synthesize 3-armed poly(γ-benzyl-l-glutamic acid) (3-PBLG) [43–45] (Fig. 1a). l-glutamic acid γ-benzyl ester *N*-carboxyanhydride (BLG NCA) was prepared following the reported protocol [44, 46]. The initiator and BLG NCA were dissolved in anhydrous THF separately with concentrations of 6.6 × 10^–3^ M and 0.262 M, respectively, in a glovebox. The mole ratio of the initiator to BLG NCA was 1:60. The NCA solution was added to the initiator solution after the completion of dissolution. The reaction mixture was stirred continuously under a nitrogen atmosphere at room temperature for 3 days. Ethyl ether was used to precipitate the final product. The 3-PBLG was collected after removing the solvents using a centrifuge, the product was washed with ethyl ether three times, and dried completely with a vacuum system (yield of 90%). 3-PBLG was prepared in TFA-*d*_*1*_ for proton nuclear magnetic resonance (^1^H NMR) measurement on an AVANCE III HD 600 NMR.

#### Debenzylation of 3-PBLG

3-armed poly(l-glutamic acid (3-PLG) nanocarriers were synthesized by using trimethylsilyl iodide (TMSI) to debenzylate 3-PBLG (Fig. 1a) [44, 47]. In a glovebox, 3-PBLG (1.0 g) was completely dissolved in anhydrous DCM (100 ml) in a round bottom flask at room temperarture. Under a dark condition, TMSI (fivefold mole amount of BLG) was added into the 3-PBLG solution for the removal of benzyl groups on PBLG segments. The reaction flask was sealed, covered by aluminum foil to prevent the light, taken out from the glovebox, and stirred at 40 °C in an oil bath for 24 h. The debenzylated product was precipitated in hexane, dried using a centrifuge and vacuum, dissolved in basic water, and purified by dialysis with a dialysis membrane (molecular weight cut-off 3.5 kDa) against deionized water (DIW) for three days before the lyophilization for removing water and collecting the final product. The freeze-dried sample was dissolved in deuterium oxide (D_2_O) for ^1^H NMR.

#### Functionalization of 3-PLG with l-alanine and l-glutathione

The A-GSH-targeted 3-PLGs (3-PLG-A-GSH) were synthesized by grafting A and GSH on 3-PLG using *N*-ethyl-*N*ʹ-(3-(dimethylamino)propyl)carbodiimide/N-hydroxysuccinimide (EDC/NHS) coupling reaction in DIW (Fig. 1b) [44, 47, 48]. The mole ratio of PLG:A:GSH was set as 1:0.3:0.3. The reaction solution was dialyzed against DIW and lyophilized to purify and collect the 3-PLG-A-GSH. The sample was prepared in D_2_O for ^1^H NMR.

#### Drug coupling on 3-PLG and 3-PLG-A-GSH

EDC/NHS coupling chemistry was used to couple dopamine and ibuprofen with 3-PLG and 3-PLG-A-GSH (Fig. 1b) [44, 47, 48]. Dopamine was grafted without structural modification in DIW whilst ibuprofen was modified with 2,2′-(ethylenedioxy)bis(ethylamine) (EG_2_-diamine) with EDC/NHS in ethanol (Fig. 1c) before grafting on the copolypeptides in DIW. The mole amount of dopamine and ibuprofen was set as 50% of polypeptide. Nanocarriers containing dopamine (3-PLG-dopa, 3-PLG-dopa-A-GSH) and ibuprofen (3-PLG-ibu, 3-PLG-ibu-A-GSH) were purified by dialysis against DIW and collected by lyophilization. All samples were labeled with *N*-(2-aminoethyl) rhodamine 6G-amide bis(trifluoroacetate) (R6G) by using the EDC/NHS coupling reaction with a weight ratio of polypeptide to R6G at 20:1 (Fig. 1b). Before the use in cellular assays the ibuprofen-coupled samples were dissolved in etanol:DIW (1:1) and dialyzed against distilled water (DW; 48 h) in D-Tube™ Dialyzer Maxi (cut off 3.6 kDa; Novagen).

### Physico-chemical characterization of nanocarriers

The average size, polydispersity index (PDI) and surface charge (zeta potential) of nanocarriers were measured by dynamic light scattering (Malvern Zetasizer Nano ZS, equipped with a He–Ne laser (λ = 632.8 nm), Malvern Instruments, UK). For measurements samples were diluted in filtered PBS to a final concentration of 1 mg/ml. Means were calculated from the average of at least 3 × 13 measurements per sample. The morphology of nanocarrier formulations was investigated under a transmission electron microscope (JEM-1400 Flash, JEOL Ltd., Japan). Preparation of samples and transmission electron microscopy imaging were done as previously described [24].

### Quantification and visualization of cellular uptake of nanocarriers

For the cellular uptake experiments hECs were cultured in 24-well plates (2 × 10^4^ cells/well, Corning Costar) coated with collagen type IV and fibronectin in 50% PC-conditioned medium. At day 5 the confluent cell layers were supplemented with cARLA cocktail. Two days later the hECs were incubated with nanocarriers (100 µg/ml) diluted in hEC medium at 37 °C for 1, 4 or 24 h in a CO_2_ incubator. To reveal the role of targeting ligands in the cellular uptake processes, alanine and GSH ligands were added at high concentration (5 mM each) in co-treatment with the targeted nanocarriers. To study the mechanisms of nanocarrier uptake, hECs were pretreated with endocytosis inhibitor randomly methylated β-cyclodextrin (CD; 5 mM; CycloLab Ltd., Hungary) or cytochalasin D (CytoD; 0.125 μg/ml) for 1 h then co-incubated with inhibitors and nanocarriers for 4 h. To investigate active metabolism hECs were co-incubated with metabolic inhibitor sodium azide (1 mg/ml) and nanocarriers at 37 °C for 4 h. To make the hEC surface charge more positive the glycocalyx of hECs was digested with neuraminidase enzyme (1 U/ml, 1-h pretreatment) or cultures were treated with cationic lipid 1-(4-trimethylammoniumphenyl)-6-phenyl-1,3,5-hexatriene (TMA-DPH; 54 µM, 30-min pretreatment; Thermo Fischer Scientific) before the 4-h uptake assay of nanocarriers. At the end of all incubations, the hECs were washed three times with ice-cold PBS containing 0.1% BSA, once with acid stripping buffer (glycine 50 mM, NaCl 100 mM, pH 3) to remove the non-internalized, but cell surface-attached nanocarriers, and finally with PBS. At the end of the experiment hECs were lysed in DW supplemented with Triton X-100 detergent (10 mg/ml). The fluorescence of R6G labeled nanocarriers in the cell lysates were measured by spectrofluorometer (Fluorolog 3, Horiba Jobin Yvon, USA) at 525 nm excitation and 551 nm emission wavelengths. The nanocarrier amount was normalized to the protein concentration in the samples measured by BCA protein assay (Thermo Fischer Scientific).

To visualize the cellular uptake hECs were cultured in 96-well black plates with clear bottom (7 × 10^3^ cells/well; Thermo Fischer Scientific; collagen IV and fibronectin coating) to protect the neighboring samples from photobleaching. The cells were differentiated with PC-conditioned medium and cARLA (48 h) and the confluent monolayers were incubated with nanocarriers (100 µg/ml) diluted in hEC culture medium at 37 °C for 24 h in a CO_2_ incubator. To stain cell nuclei Hoechst 33342 dye (1 μg/ml, 10 min) was used. After the incubation, hECs were washed with Ringer-HEPES buffer (118 mM NaCl, 4.8 mM KCl, 2.5 mM CaCl_2_, 1.2 mM MgSO_4_, 5.5 mM d-glucose, 20 mM HEPES, pH 7.4) supplemented with 1% FBS. After the washing step the R6G dye of the internalized non-targeted or targeted nanocarriers in living hECs was imaged using the 543 nm laser line on a Leica TCS SP5 confocal laser scanning microscope equipped with a sample holder kept at 37 °C. Image analysis based on fluorescence intensity was performed as described in our previous study [24].

### Permeability assays

To establish the co-culture BBB model brain PCs were passaged at P11 (7 × 10^3^ cells/insert) to the bottom (brain) side of culture inserts (polyester membrane; pore size: 0.4 μm; surface: 0.33 cm^2^; Corning Costar; collagen type IV and fibronectin coating). After the attachment of PCs (3 h; 37 °C in a CO_2_ incubator) hECs (P6) were seeded (2 × 10^4^ cells/insert) to the upper (blood) side of the membranes. Then, the inserts containing cells were placed into 24-well plates containing endothelial culture medium. The two cell types were cultured together for 7 days and the model was treated with cARLA (48 h) before the permeability assays. The barrier tightness of the model was verified by transendothelial electrical resistance (TEER) measurements using an EVOM voltohmmeter (World Precision Instruments, USA) combined with STX-2 electrodes. Values were calculated using geometric correction factor and insert diameter as described in our previous study [49]. When TEER values reached a plateau level (82 ± 2 Ω × cm^2^; n = 30), the model was used for experiments.

Nanoparticles were diluted in phenol red-free DMEM/HAM’s F-12 medium (Gibco, Life Technologies, Carlsbad, CA, USA) supplemented with 5% FBS at a final concentration of 100 µg/ml and added to the donor compartment (0.2 ml). The model was incubated at 37 °C for 24 h on a horizontal shaker (150 rpm) in a CO_2_ incubator. To measure the integrity of the model, the paracellular marker sodium fluorescein (SF; 376 Da, 10 μg/ml) and the transcellular marker Evans blue-albumin complex (EBA; 67 kDa, 10 mg/ml BSA + 167.5 μg/ml Evans blue) were also tested for permeability. After incubation, samples were collected from the acceptor compartments and the fluorescent signal of R6G labeled nanocarriers (excitation: 525 nm; emission: 551 nm) was quantified with a spectrofluorometer (Fluorolog 3, Horiba Jobin Yvon). The concentration of the marker molecules was quantified at 485 nm excitation and 515 nm emission wavelengths (SF) and 584 nm excitation and 663 nm emission wavelengths (EBA). The apparent permeability coefficients (P_app_) were calculated as described previously [40] with the following equation:

$$\frac{\Delta {\left[C\right]}_{A} \times {V}_{A}}{ A \times {\left[C\right]}_{D} \times \Delta t},$$

where Δ[C]_A_ is the concentration difference of in the acceptor compartment at 24 h, [C]_D_ is the concentration in the donor compartment at 0 h, V_A_ is the volume of the acceptor compartment (0.9 ml), and A is the surface area available for permeability (0.33 cm^2^).

The pore size of cell culture inserts is an important parameter and we compared cell culture inserts with 3 µm and 0.4 µm pore sizes (Corning Costar; 0.33 cm^2^) in a preliminary permeability test. As shown in Fig. S1, 3-PLG-ibu-A-GSH nanocarriers penetrated across the BBB model on both insert types. Although the permeability value of targeted nanocarriers were lower in case of BBB models on the inserts with 0.4 µm pore size membranes, the significant difference between the non-targeted and targeted groups remained the same and the penetration of marker molecules across the BBB model on inserts with 3 µm pore-size membranes or 0.4 µm pore-size membranes were also similar. Based on these results all further permeability assays were made on inserts with 0.4 µm pore size membranes.

In addition to the quantification of R6G, we made efforts to determine the concentration of dopamine and ibuprofen from the samples of the permeability experiments by liquid chromatography-tandem mass spectrometry. The lower limit of quantification (LLOQ) was 50 ng/ml for ibuprofen with signal-to-noise (S/N) ratios > 10. The lower limit of quantification (LLOQ) for dopamine was 0.5 μg/ml, however, serum and nutrients in the permeability medium did not allow its quantification. Despite the optimization of measurement protocols (see in the Additional file), the quantification of the active agents was unsuccessful.

### Nanocarrier uptake in midbrain organoids

Control or PD organoids were embedded in 10 μl Geltrex (Thermo Fischer Scientific) at the bottom of 24-well plates (1 organoid/well), then culture inserts were placed above them containing the BBB model as described in our previous study [24]. Donor compartments received 3-PLG-dopa or 3-PLG-dopa-A-GSH (100 µg/ml) diluted in phenol red-free DMEM/HAM’s F-12 medium supplemented with 5% FBS and the model was incubated for 24 h. At the end of the permeability assay midbrain organoids were removed from the bottom of acceptor compartments, homogenized in DW containing Triton X-100 (10 mg/ml). Samples were centrifuged (13,000 rpm, 1 min, Biofuge Pico, Heraeus, Thermo Fisher Scientific, USA) and the fluorescent signal of nanocarriers was quantified from the supernatant with spectrofluorometer. The protein concentration of the samples was measured by BCA protein assay. The nanocarrier uptake was normalized to the protein content of organoids.

The plastic bottom of standard 24-well plates (Corning Costar, USA) was replaced by borosilicate glass coverslips (VWR International, USA). The plates were sterilized and midbrain organoids were embedded in a Geltrex drop. Inserts with hECs and PCs were placed into the wells above the organoids and dopamine-coupled nanoparticles were added in the donor compartments. In the last 2 h of the 24-h permeability assays, the acceptor compartments were supplemented with Hoechst 33342 dye (2 μg/ml) to label the cell nuclei of organoids. The inserts were removed, the wells were washed with phenol red-free DMEM/HAM’s F-12 medium supplemented with 5% FBS and the uptake of nanocarriers in the organoids was immediately visualized by confocal laser scanning microscope (Olympus Fluoview FV1000, Olympus Life Science Europa GmbH, Germany).

### Cytokine treatment

The possible protective effect of ibuprofen-coupled nanocarriers against cytokine-induced barrier damage was measured by real-time impedance kinetics. Plates with integrated gold electrodes (E-plate 96; Agilent Technologies, USA) were coated with collagen IV and fibronectin and the cell-free background was measured in culture medium for each well by an xCELLigence RTCA SP device (Agilent Technologies). Then, hECs were seeded in the plates at a density of 7 × 10^3^ cells/well in endothelial medium containing 50% PC-conditioned medium. At day 5 the cells received cARLA (48 h) to induce brain-like properties. To induce barrier damage cells were incubated with proinflammatory cytokines (CK) tumor necrosis factor-α (TNF-α) and interleukin-1β (IL1-β) both at 10 ng/ml concentration. Cells were also treated with ibuprofen (0.9 and 1.5 µM) and nanocarriers 3-PLG-ibu or 3-PLG-ibu-A-GSH (100 µg/ml). The impedance of cells was followed at 10 kHz at every 5 min for 24 h. The cell index was defined as R_n_-R_b_ at each time point of measurement, where R_n_ is the cell-electrode impedance of the well when it contains cells, and R_b_ is the background impedance of the well with the medium alone. The cell index was normalized in each well to the value measured at the last time point before the treatment.

To test the protective effects of nanocarriers against CK-induced permeability changes the donor compartments were treated with CK during the 24-h permeability assays. Ibuprofen (1.5 µM) or 3-PLG-ibu and 3-PLG-ibu-A-GSH nanocarriers (100 µg/ml) were added as co-treatments. At the end of the 24-h incubation, samples from the donor and the acceptor compartments were collected, then the permeability of SF and EBA markers was tested for 30 min. The fluorescent intensity of nanocarriers and markers was determined from the samples by spectrofluorometry and P_app_ values were calculated.

### Statistics

Data are presented as means ± SD. Statistical analyses were performed using GraphPad Prism 8 software (GraphPad Software, USA). Means were compared using Student’s t-test, one-way or two-way ANOVA followed by Dunnett’s or Tukey’s post-test. Differences were considered statistically significant at p < 0.05. All experiments were repeated at least two times, and the number of parallel samples in each experiment was 4–10.

## Results

### Human BBB co-culture model

**Fig. 2:**
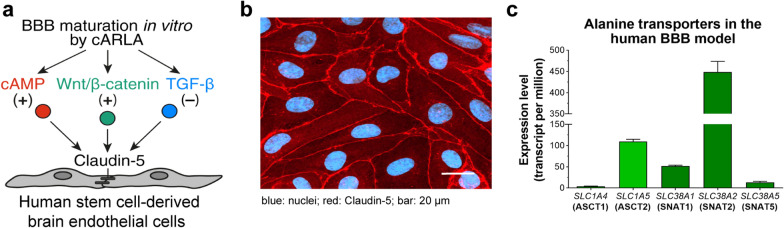
The human BBB co-culture model. **a** Signaling pathways related to BBB maturation induced by cARLA. **b** Claudin-5 immunostaining of human stem cell-derived brain endothelial cells. Blue: nuclei; red: claudin-5, scale bar: 20 µm. **c** Expression levels of genes encoding alanine transporters in the cARLA treated human BBB model. Values presented are means ± SD

In this study, a stem cell-derived human BBB model was used. To tighten barrier integrity and induce BBB properties, a cocktail of small molecules, cARLA, was applied to activate the cyclic AMP and Wnt/β-catenin and inhibit the TGF-β signaling pathways (Fig. 2a) [40]. The 48-h cARLA treatment resulted in mature brain endothelial cell morphology with continuous and strong claudin-5 junctional protein immunostaining at the cell borders (Fig. 2b). Brain microvascular endothelial cells express five carriers for the amino acid alanine belonging to two groups, neutral amino acid transporters (ASCT/SLC1A) or small neutral amino acid transporters (SNAT/SLC38A). From these the *SNAT2*/*SLC38A2* showed the highest expression in our model (Fig. 2c). The *ASCT2/SLC1A5* and *SNAT1/SLC38A1* genes were expressed at moderate levels, while the genes of *ASCT1/SLC1A4* and *SNAT5/SLC38A5* were at low levels in brain endothelial cells.

### Characterization of BBB-targeted nanocarriers

The successful synthesis of the co-polypeptides, the degree of polymerization, and the grafting ratios were determined by ^1^H NMR (Fig. S2-S3, Table S1a). The nanocarriers were synthesized as shown in Fig. 1. The degree of polymerization of 3-PBLG was calculated by the ratio of the integrated areas of proton d on benzyl groups of PBLG and proton b on the initiator (Fig. S2a, Table S1a). After the removal of benzyl groups, the successful deprotection was confirmed with the percentage of the remaining benzyl groups lower than 5% based on the ^1^H NMR data (Fig. S2b). EDC/NHS chemistry was used to graft functional groups (alanine and GSH), couple therapeutic molecules (ibuprofen and dopamine) on PLG backbone and label the co-polypeptides with R6G (Fig. 1c). Based on ^1^H NMR, the grafting ratio of alanine and GSH were 0.027 and 0.13, respectively, calculated by the ratio of the integrated areas of proton f on alanine and proton l on GSH (Fig. S2c, Table S1a). After drug coupling, the grafting ratio of ibuprofen and dopamine was determined based on ^1^H NMR as shown in Fig. S3 and Table S1a. All the results of the analytical characterization are summarized in Fig. S2-4; Table S1a.

**Fig. 3:**
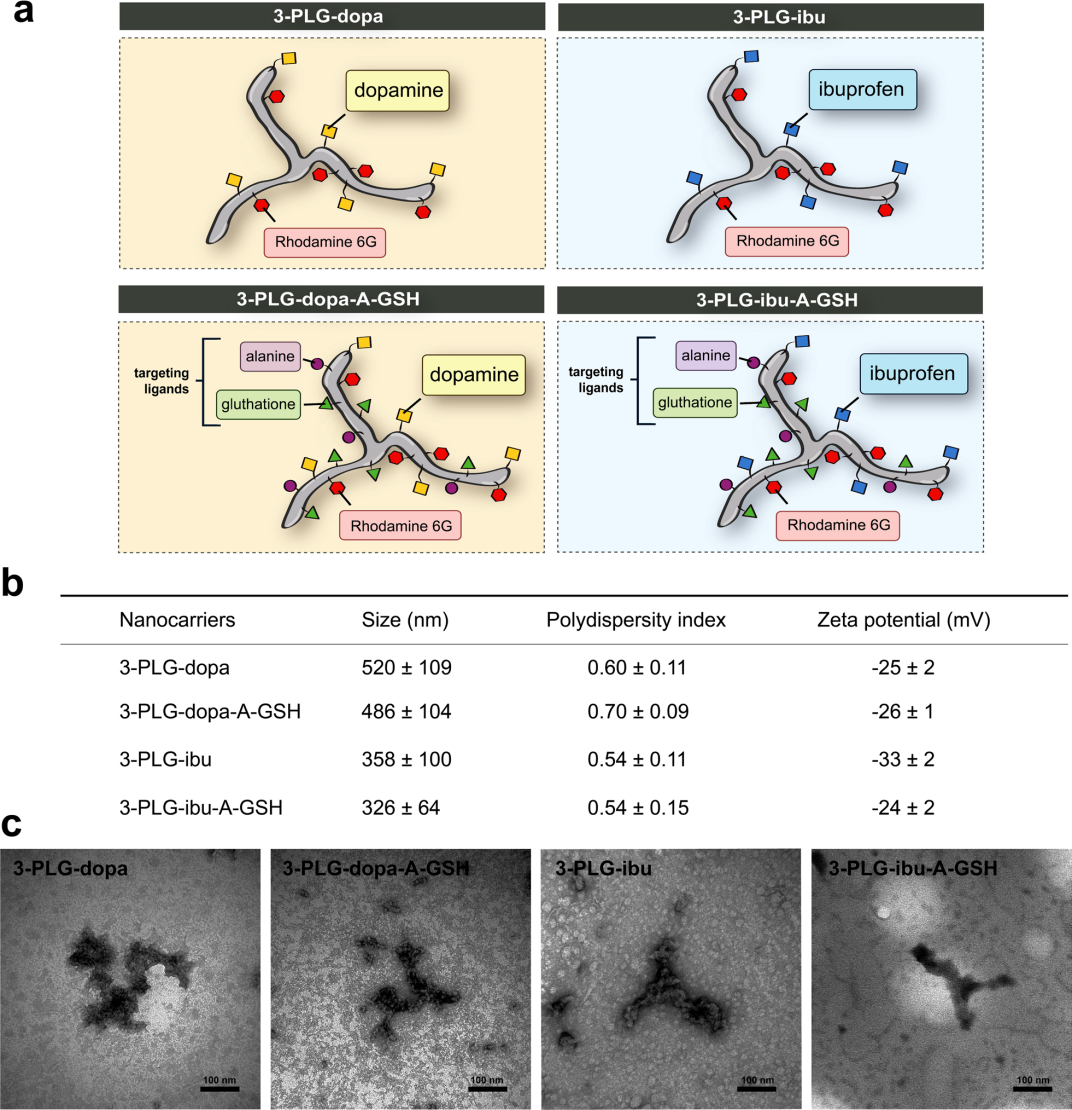
Characterization of nanocarriers. **a** Schematic drawing of non-targeted, 3-armed poly(L-glutamic acid) nanocarriers grafted with dopamine (3-PLG-dopa) or ibuprofen (3-PLG-ibu) and their L-alanine (A) and glutathione (GSH) dual-targeted (3-PLG-dopa-A-GSH; 3-PLG-ibu-A-GSH) formulations. The copolypeptides were labeled by rhodamine 6G (R6G). **b** The main physico-chemical properties of nanocarriers. Values presented are means ± SD. **c** Transmission electron microscopy images of nanoformulations. Scale bar: 100 nm

Schematic drawings of non-targeted and A-GSH dual-targeted nanoformulations are presented in Fig. 1b and Fig. 3a. The mean diameter of 3-armed nanocarriers was between 326 and 520 nm (Fig. 3b) by dynamic light scattering measurements. The size distribution of nanocarriers was relatively wide as indicated by PDI values between 0.5 and 0.7 in all groups (Fig. 3b). It should be noted, however, the dynamic light scattering technique is not well-suited for size determination of branching nanoparticles, since assembly or aggregation of the polypeptide carriers cannot be excluded. The zeta potential of all nanocarriers was negative (≤ − 24 mV) (Fig. 3b).

The nanocarriers showed filamentous, branched structure by transmission electron microscopy (Fig. 3c). The nanoparticle groups exhibited similar shape but the structure of polypeptides is highly influenced by the sample preparation steps (treatment with ethanol, drying steps) for electron microscopy imaging.

After the synthesis of the nanocarriers, the samples were lyophilized. The stocks were always freshly dissolved and immediately used in the cellular assays. There was no obvious trend for change neither in the size nor in the PDI values of diluted nanocarriers stored at 4 °C for 6 months (Table S1b). Based on these measurements, the size of dissolved nanoparticle samples were relatively stable even after six months, but aggregation or disintegration cannot be excluded due to the large PDI values.

### Cellular uptake of nanocarriers

**Fig. 4:**
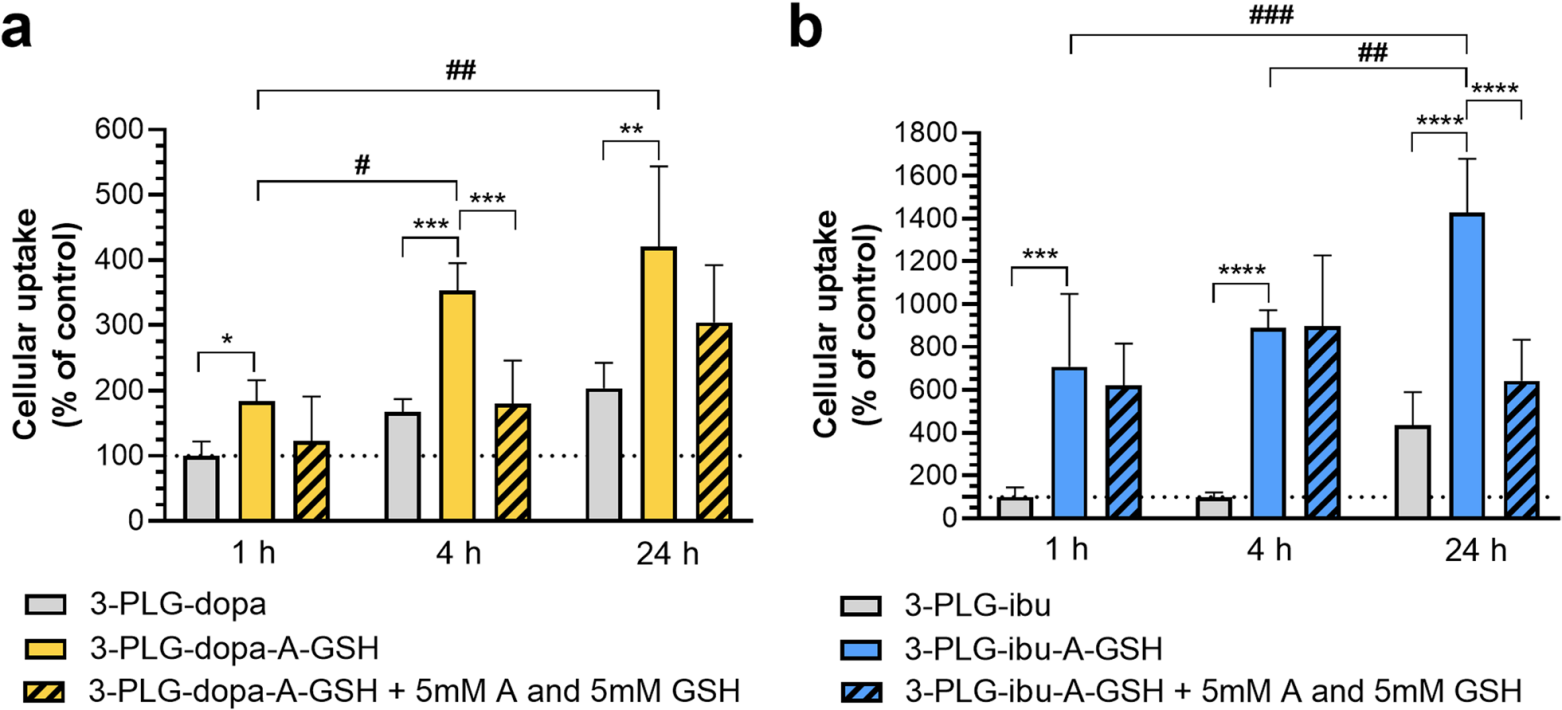
Cellular uptake of **a** dopamine- (3-PLG-dopa; 3-PLG-dopa-A-GSH) or **b** ibuprofen-coupled (3-PLG-ibu; 3-PLG-ibu-A-GSH) nanocarriers in brain endothelial cells after 1, 4 and 24 h of incubation (100 µg/ml; 37 °C) and the effect of free l-alanine and glutathione (GSH) ligands (5 mM each in co-treatment with nanocarriers) on the cellular internalization of dual-targeted nanocarriers. Values presented are means ± SD and given as a percentage of the 3-PLG-dopa or 3-PLG-ibu groups at 1 h-time point. Statistical analysis: two-way ANOVA, Tukey’s post-test; *p < 0.05; **p < 0.01; ***p < 0.001; ****p < 0.0001 compared to the 3-PLG-dopa-A-GSH or 3-PLG-ibu-A-GSH groups at each time-points; ^#^p < 0.05; ^##^p < 0.01; ^###^p < 0.001 between the 3-PLG-dopa-A-GSH or 3-PLG-ibu-A-GSH groups at each time point; n = 6

The incubation of brain endothelial cells with nanocarriers did not decrease the impedance of monolayers reflecting good cell viability (Fig. S5-6). The brain endothelial internalization of targeted nanocarriers 3-PLG-dopa-A-GSH and 3-PLG-ibu-A-GSH was significantly higher at all time-points compared to the non-targeted groups (Fig. 4a, b). A time-dependent increase could be observed which was more pronounced in the targeted nanocarriers groups. The highest uptake levels for both 3-PLG-dopa-A-GSH and 3-PLG-ibu-A-GSH were measured at the 24-h time-point: a significant, more than twofold elevation was obtained compared to the 1-h targeted groups (Fig. 4a, b). The uptake values for the nanocarriers at the 1-h time-point were 3.4 ng/µg protein in the non-targeted 3-PLG-dopa and 0.3 ng/µg protein in the 3-PLG-ibu groups (Fig. 4).

To verify the role of targeting ligands in the cellular uptake process, alanine and GSH were added at high concentrations during the uptake assay of the targeted nanocarriers. Treatments with free ligands at 5 mM concentration had not only no toxic effects but even improved the impedance of the cell layers (Fig. S7). The addition of alanine and GSH ligands inhibited the internalization of targeted nanocarriers (Fig. 4a, b). These changes were significant at 4-h and 24-h time-points for PLG-dopa-A-GSH and 3-PLG-ibu-A-GSH, respectively.

**Fig. 5:**
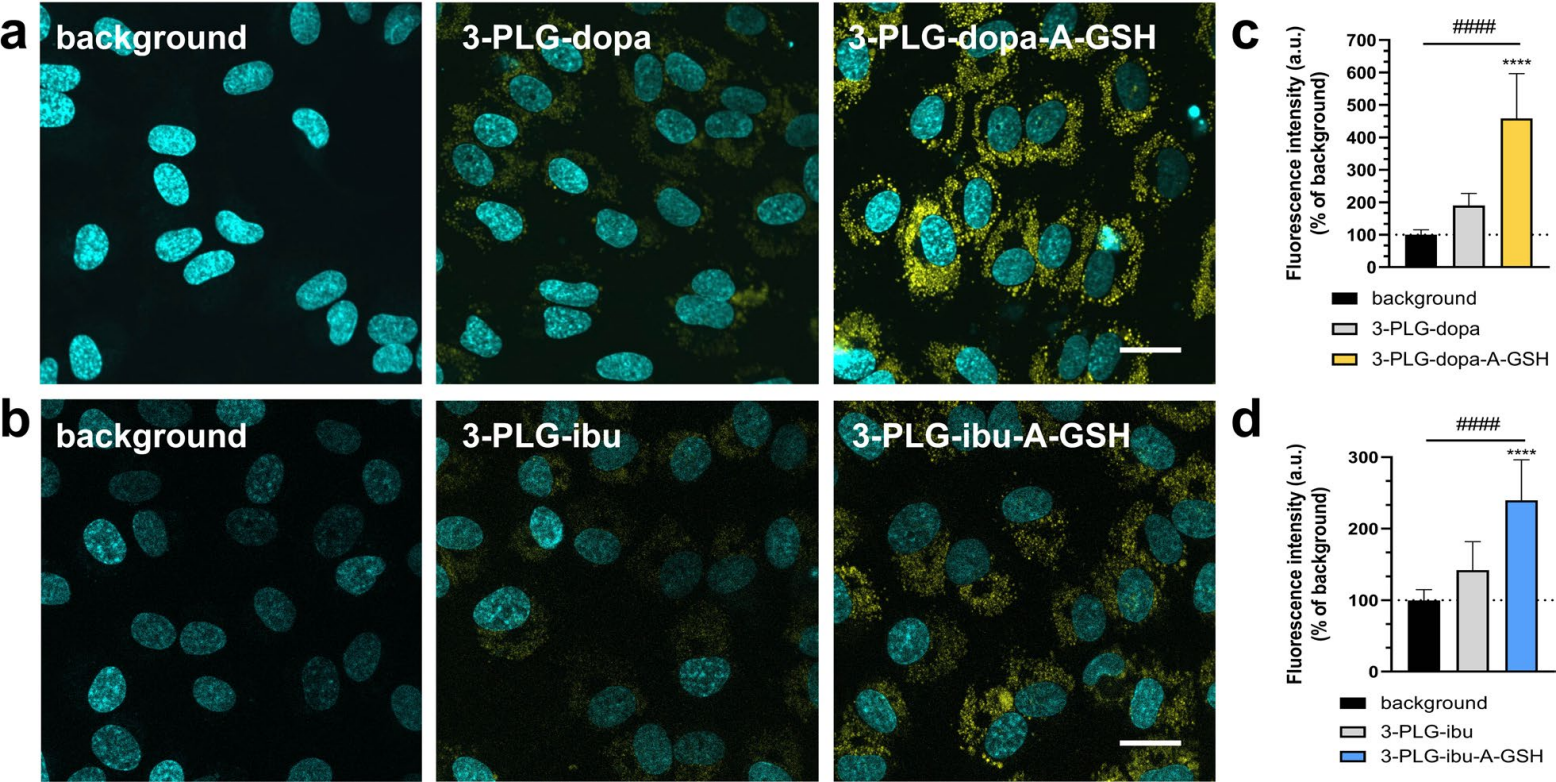
Live cell visualization of cellular uptake. Confocal microscopy images of living brain endothelial cells after 24-h incubation with** a** dopamine- (3-PLG-dopa; 3-PLG-dopa-A-GSH) or **b** ibuprofen-coupled (3-PLG-ibu; 3-PLG-ibu-A-GSH) nanoparticles. Nanocarriers: yellow; cell nuclei: cyan; scale bar: 20 μm. Image analysis of cellular entry of **c** dopamine- or **d** ibuprofen-coupled nanocarriers. Values are presented as means ± SD, and shown as percentage of the untreated background fluorescent intensity given as arbitrary units (a.u.). Statistical analysis: one-way ANOVA, Tukey’s post-test; ****p < 0.0001 compared to the 3-PLG-dopa or 3-PLG-ibu groups; ^####^p < 0.0001 compared to the background; n = 4–10

The live confocal microscopy images verified the high-level vesicular uptake of the dual-targeted nanocarriers compared to the non-targeted groups in brain endothelial cells (Fig. 5a, b). Low fluorescent R6G signal could be detected in the cytoplasm of brain endothelial cells that received 3-PLG-dopa or 3-PLG-ibu as compared to the high signal intensive in the dual-targeted dopamine- or ibuprofen-coupled nanocarrier groups (Fig. 5a, b). In concordance with the results of the uptake experiments, the image analysis also verified that the cellular internalization of targeted nanocarriers was significantly higher (2.4-times elevation for 3-PLG-dopa-A-GSH; 1.7-times elevation for 3-PLG-ibu-A-GSH) than the uptake in the non-targeted groups (Fig. 5c, d).

To detect the intracellular fate of the dopamine- (3-PLG-dopa; 3-PLG-dopa-A-GSH) or ibuprofen-coupled (3-PLG-ibu; 3-PLG-ibu-A-GSH) nanocarriers we performed co-localization imaging with the Golgi apparatus in living brain endothelial cells (Fig S8). Nanocarriers, especially the targeted ones, could be visualized in the cytoplasm of cells but not in Golgi (Fig S8a). Based on image analysis, the co-localization area of Golgi and nanocarriers was limited (Fig S8b).

**Fig. 6:**
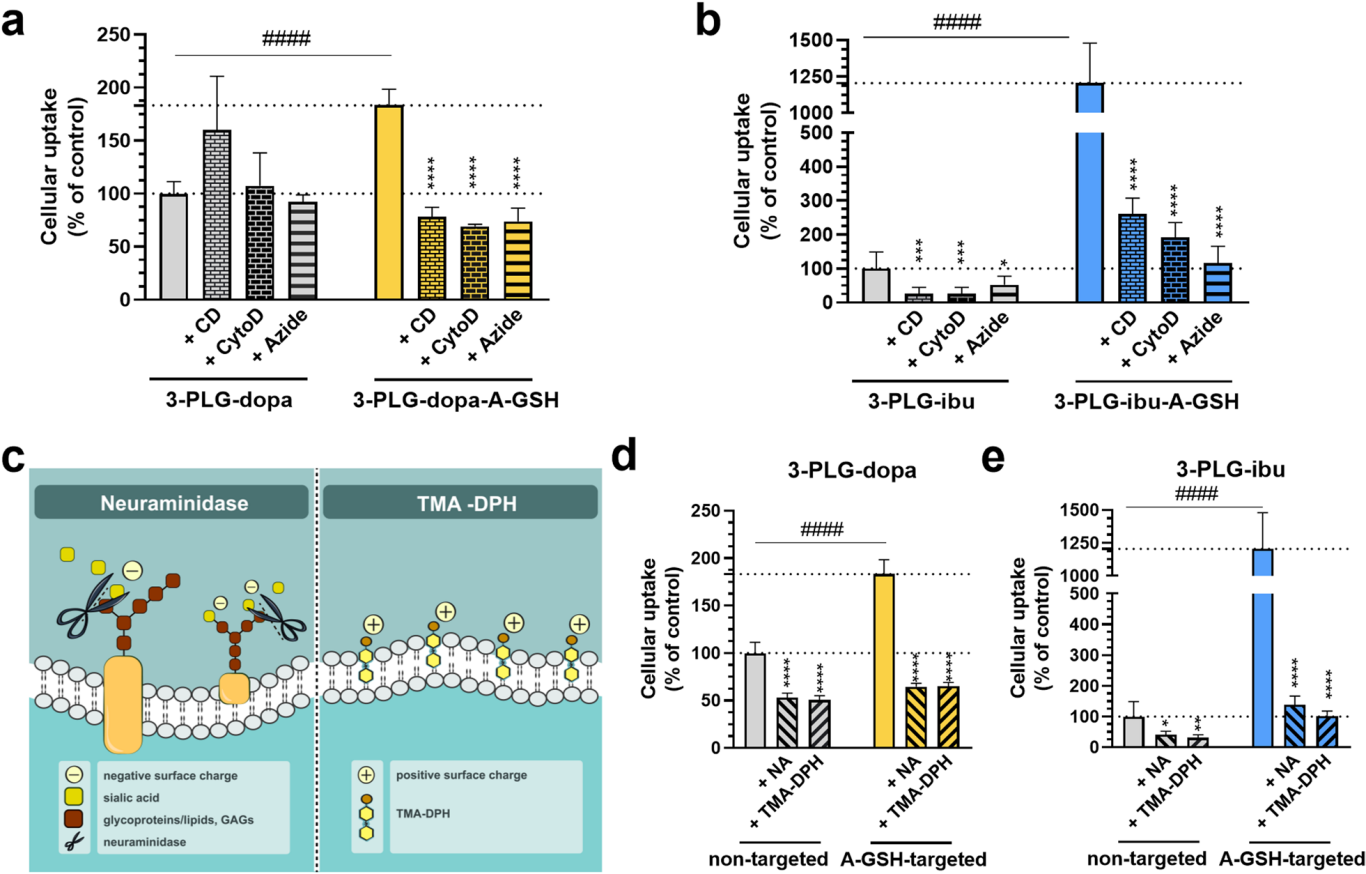
Mechanisms of nanocarrier cell entry. The effects of endocytosis inhibitors randomly methylated β-cyclodextrin (CD) or cytochalasin D (CytoD) and metabolic inhibitor sodium azide on the uptake of **a** 3-PLG-dopa and 3-PLG-dopa-A-GSH, and **b** 3-PLG-ibu and 3-PLG-ibu-A-GSH nanocarriers. **c** Schematic drawing of the modification of brain endothelial surface charge by neuraminidase (NA) enzyme or cationic lipid TMA-DPH. The effect of NA and TMA-DPH on the cellular uptake of **d** 3-PLG-dopa and 3-PLG-dopa-A-GSH and **e** 3-PLG-ibu and 3-PLG-ibu-A-GSH nanocarriers. Values presented are means ± SD and are given as a percentage of the non-targeted nanoparticle groups. Statistical analysis: two-way ANOVA, Dunnett post-test; *p < 0.05; **p < 0.01; ***p < 0.001; ****p < 0.0001 compared to the non-treated control in each groups; ^####^p < 0.0001 compared to non-targeted groups. n = 4–6

Before nanocarrier cell entry experiments, the non-toxic concentrations of endocytosis and metabolic inhibitors on the cARLA treated hEC were determined by cell viability assays, namely impedance measurement and MTT tests (Fig. S9). Endocytosis inhibitors randomly methylated β-cyclodextrin and cytochalasin D reduced the uptake of dopamine- (3-PLG-dopa-A-GSH) or ibuprofen-coupled (3-PLG-ibu; 3-PLG-ibu-A-GSH) nanocarriers (Fig. 6a, b). Both inhibitors decreased the cellular internalization of targeted nanoparticles to less than half as compared to the baseline levels of the control groups (Fig. 6a, b). These data indicate that the uptake mechanism of nanocarriers was mediated by endocytosis. The metabolic inhibitor sodium azide also significantly reduced the cellular uptake of the nanocarriers, suggesting an active cellular process (Fig. 6a, b). The uptake values for the nanocarriers at the 4-h time-point were 3.2 ng/µg protein in the non-targeted 3-PLG-dopa and 0.4 ng/µg protein in the 3-PLG-ibu groups (Fig. 6).

The effect of surface charge on the uptake of nanocarriers was revealed by the modification of the negatively charged brain endothelial cell surface (Fig. 6c). Digestion of the sialic acid residues from the glycocalyx by neuraminidase enzyme or incubation of the cells with cationic lipid TMA-DPH made the charge of human brain endothelial cells more positive. These modifications significantly inhibited the cellular uptake of both dopamine- or ibuprofen-coupled nanocarriers compared to the unmodified control groups (Fig. 6d, e).

### Permeability of nanocarriers across the human BBB co-culture model

**Fig. 7:**
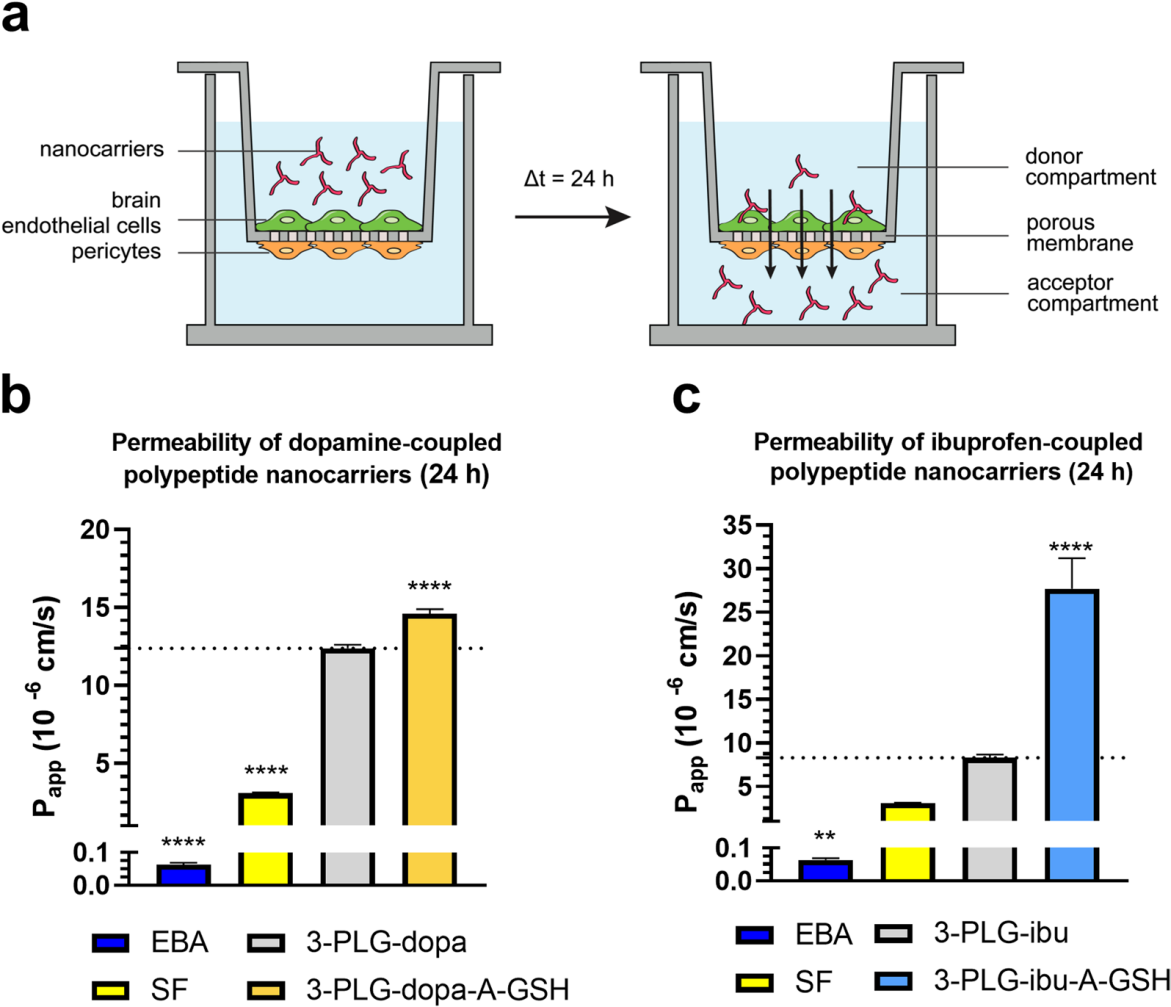
Permeability of nanocarriers across the human co-culture model of the BBB. **a** Schematic drawing of the experimental set-up. Permeability of Evans blue-albumin (EBA), sodium fluorescein (SF) reference marker molecules and **b** 3-PLG-dopa and 3-PLG-dopa-A-GSH and **c** 3-PLG-ibu and 3-PLG-ibu-A-GSH nanocarriers across the human BBB co-culture model. Values are means ± SD. Statistical analysis: one-way ANOVA followed by Dunnett test. **p < 0.01; ****p < 0.0001 compared to the 3-PLG-dopa or 3-PLG-ibu groups. n = 4–6. P_app_: apparent permeability coefficient

The permeability of the nanocarriers was investigated on a human co-culture BBB model as shown in Fig. 7a. The penetration of nanocarriers was followed from the donor (upper) to the acceptor (lower) compartment mimicking blood to brain direction. The barrier integrity of the model was verified by permeability measurements for the transcellular biomarker EBA and small hydrophilic paracellular reference molecule fluorescein. The low P_app_ values for EBA (0.06 × 10^–6^ cm/s) and fluorescein (3.08 × 10^–6^ cm/s) proves the good integrity of the co-culture BBB model (Fig. 7b, c). The non-targeted nanocarriers (3-PLG-dopa; 3-PLG-ibu) crossed the BBB model at a higher level as compared to the reference markers with low penetration (Fig. 7b, c). The dual-targeting significantly increased the permeability of 3-PLG-dopa-A-GSH (P_app_: 14.61 × 10^–6^ cm/s) compared to the non-targeted 3-PLG-dopa (P_app_: 12.39 × 10^–6^ cm/s) group (Fig. 7b). Similarly, a significant elevation was measured in the permeability of targeted ibuprofen-coupled nanocarrier 3-PLG-ibu-A-GSH (P_app_: 27.70 × 10^–6^ cm/s) compared to the non-targeted 3-PLG-ibu (P_app_: 8.29 × 10^–6^ cm/s) polypeptide in the 24-h assay (Fig. 7c). A 30-min permeability test for SF and EBA markers performed after the 24-h nanocarrier permeability assays confirmed that the integrity of the BBB models was preserved and the nanocarriers had no barrier damaging effect (Table S2). High mass balance values were obtained for all nanocarrier groups, which indicates the good recovery of the nanoparticles from the permeability samples (Table S3) and minimal sequestration in the cells or binding to the plastic surfaces of the culture-ware.

### BBB-permeability of dopamine-coupled nanocarriers and entry into midbrain organoids

**Fig. 8:**
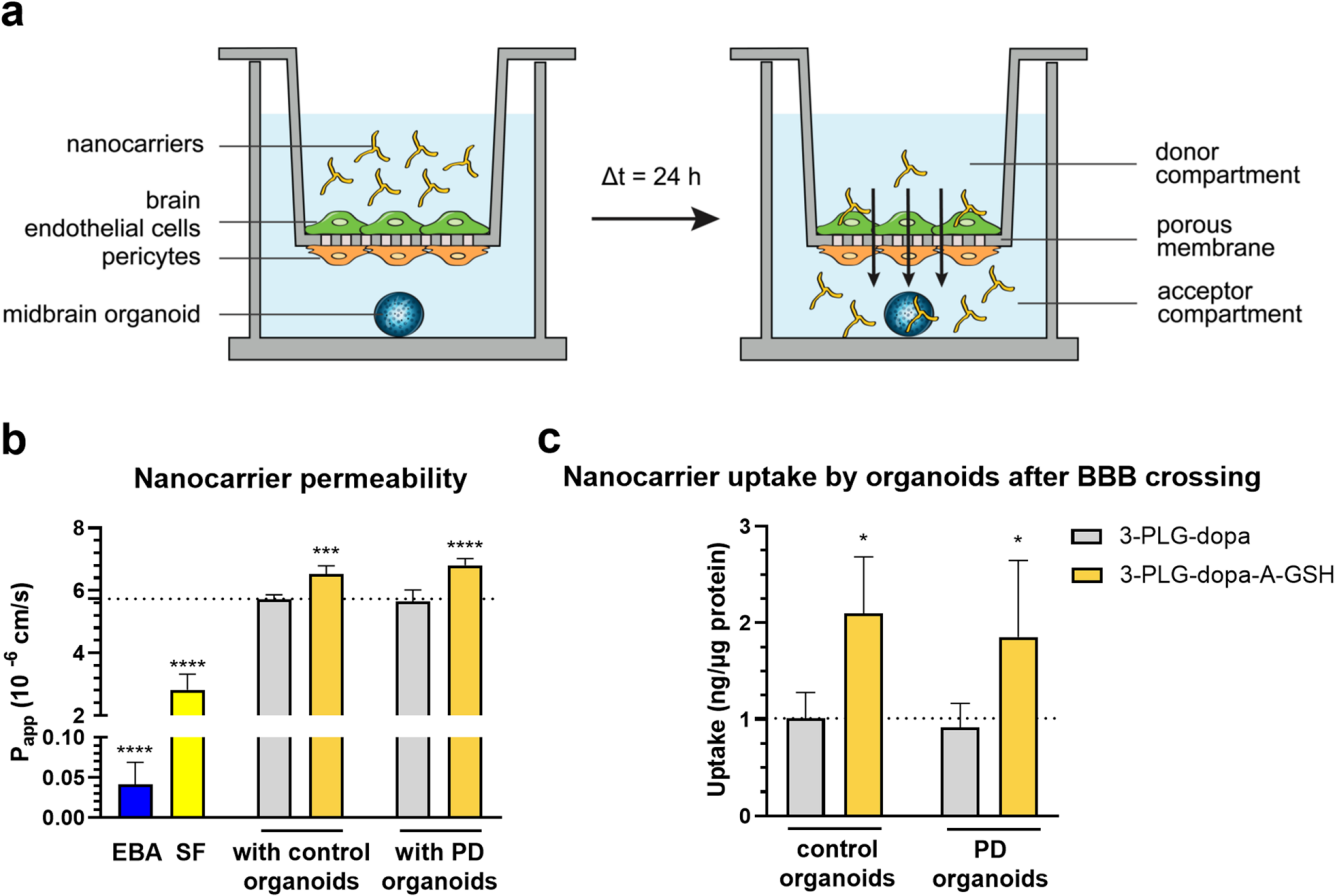
Permeability of non-targeted (3-PLG-dopa) and alanine-glutathione-targeted (3-PLG-dopa-A-GSH) nanocarriers across the human BBB co-culture model and entry into human midbrain-specific organoids. **a** Schematic drawing of the experimental set-up. **b** Permeability of dopamine coupled nanocarriers, and Evans blue-albumin (EBA) and sodium fluorescein (SF) reference marker molecules across the co-culture model in the presence of midbrain-specific organoids derived from healthy (control) and Parkinson’s disease (PD) patients’ cells. **c** Cellular uptake of nanocarriers by organoids after crossing the BBB. Values are means ± SD. Statistical analysis: two-way ANOVA, Tukey’s post-test. *p < 0.05, ***p < 0.001, ****p < 0.0001 compared to the 3-PLG-dopa data in both organoid groups. Permeability values of EBA and SF were compared to the 3-PLG-dopa group with control organoids (****p < 0.0001; one-way ANOVA, Dunnett test). n = 6 organoids/group. P_app_: apparent permeability coefficient

In this set of experiments not only the transfer of dopamine-coupled nanocarriers across the BBB model but its entry into 3D brain organoids was also studied. Midbrain-specific organoids derived from healthy (control) or PD patients’ stem cells were embedded in the bottom compartment, below the cell culture inserts (Fig. 8a). With this setup we could examine both the permeability and the subsequent nanocarrier uptake by organoids in the same experiment. The permeability of the non-targeted nanocarriers was similar in the BBB models with control (3-PLG-dopa P_app_: 5.73 × 10^–6^ cm/s) or PD organoids (3-PLG-dopa P_app_: 5.65 × 10^–6^ cm/s) in the acceptor compartment (Fig. 8b). The coupling of A-GSH targeting ligands resulted in significantly higher BBB penetrations for the 3-PLG-dopa-A-GSH nanocarriers both in the presence of organoids from healthy (3-PLG-dopa-A-GSH P_app_: 6.52 × 10^–6^ cm/s) and PD cells (3-PLG-dopa-A-GSH P_app_: 6.80 × 10^–6^ cm/s) compared to the permeability values of non-targeted formulations. There was no significant difference in the permeability of 3-PLG-dopa-A-GSH between the control and the PD organoid groups.

The barrier integrity of the BBB model remained intact for both EBA (P_app_: 0.04 × 10^–6^ cm/s) and fluorescein markers (P_app_: 2.82 × 10^–6^ cm/s) at the end of the nanocarrier permeability assay. The low P_app_ values (Fig. 8b) for the marker molecules and the continuous claudin-5 immunostaining at the cell borders of brain endothelial cells (Fig. S10) reflect that the model’s barrier integrity was not changed during the nanocarrier permeability experiment confirming the robustness of the model and the accuracy of the assay. The presence of targeting ligands resulted in two-fold higher uptake of nanocarriers in the control healthy (2.09 ng/µg protein) and PD organoids (1.85 ng/µg protein) compared to uptake of the non-targeted nanocarriers in the control (1.01 ng/µg protein) and PD (0.92 ng/µg protein) organoids (Fig. 8c). There was no difference in the uptake of the two types of nanocarriers between the healthy control and the PD organoids (Fig. 8c).

**Fig. 9:**
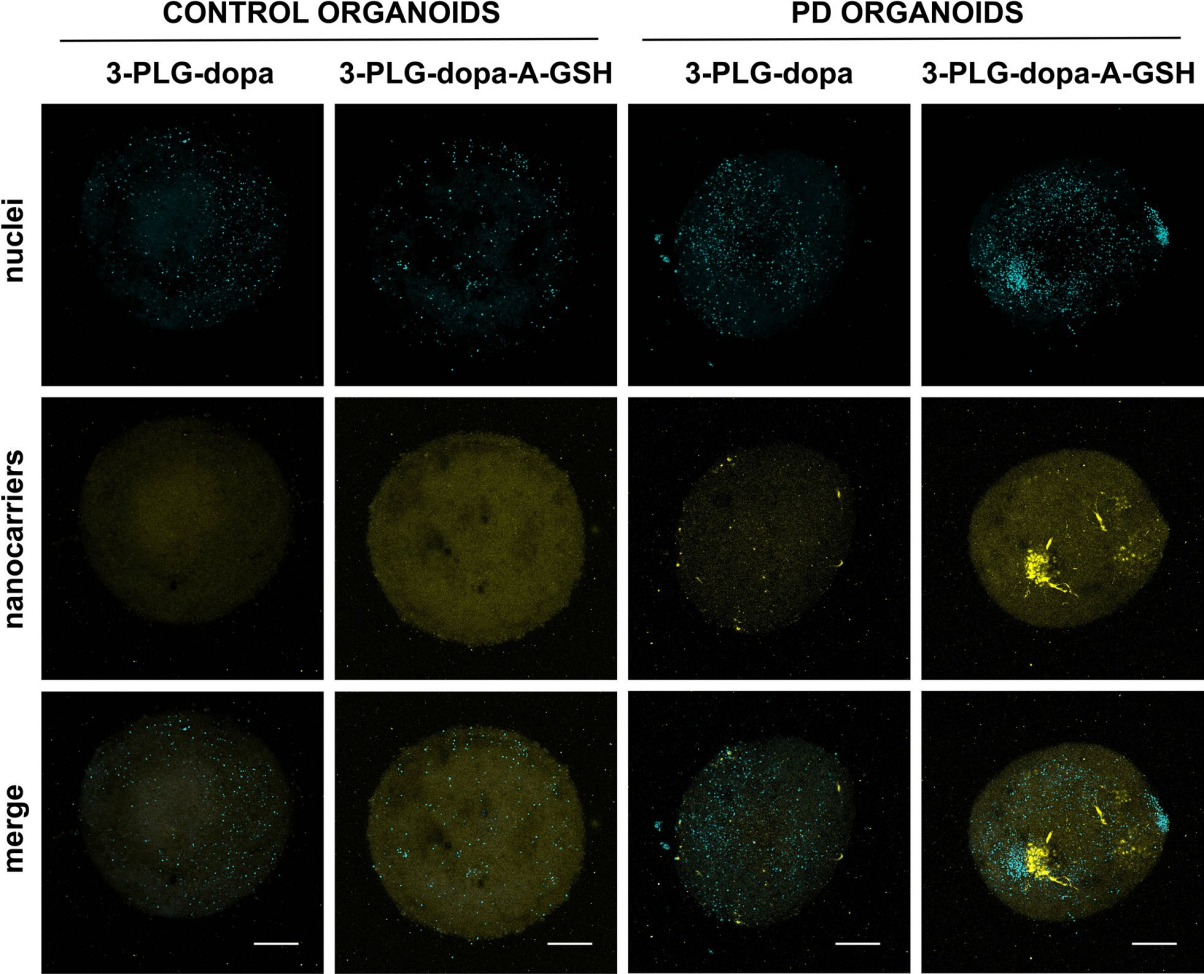
Representative confocal fluorescent microscopy images showing the uptake of non-targeted (3-PLG-dopa) and alanine-glutathione-targeted (3-PLG-dopa-A-GSH) nanocarriers (yellow) by one control and one PD midbrain-specific organoids after crossing the BBB model. Cell nuclei are stained by Hoechst 33342 (cyan). Scale bar: 200 μm

The entry of dopamine-coupled nanocarriers into living midbrain organoids was monitored by confocal microscopy (Fig. 9). In concordance with the uptake data quantified by fluorescent spectroscopy shown on Fig. 8c, more intensive R6G fluorescent signal of 3-PLG-dopa-A-GSH can be seen on the representative images showing brain organoids from healthy or PD organoids compared to the dye of non-targeted 3-PLG-dopa groups (Fig. 9).

### Protective effect of ibuprofen-coupled nanocarriers on the barrier integrity of cytokine treated human BBB model

**Fig. 10:**
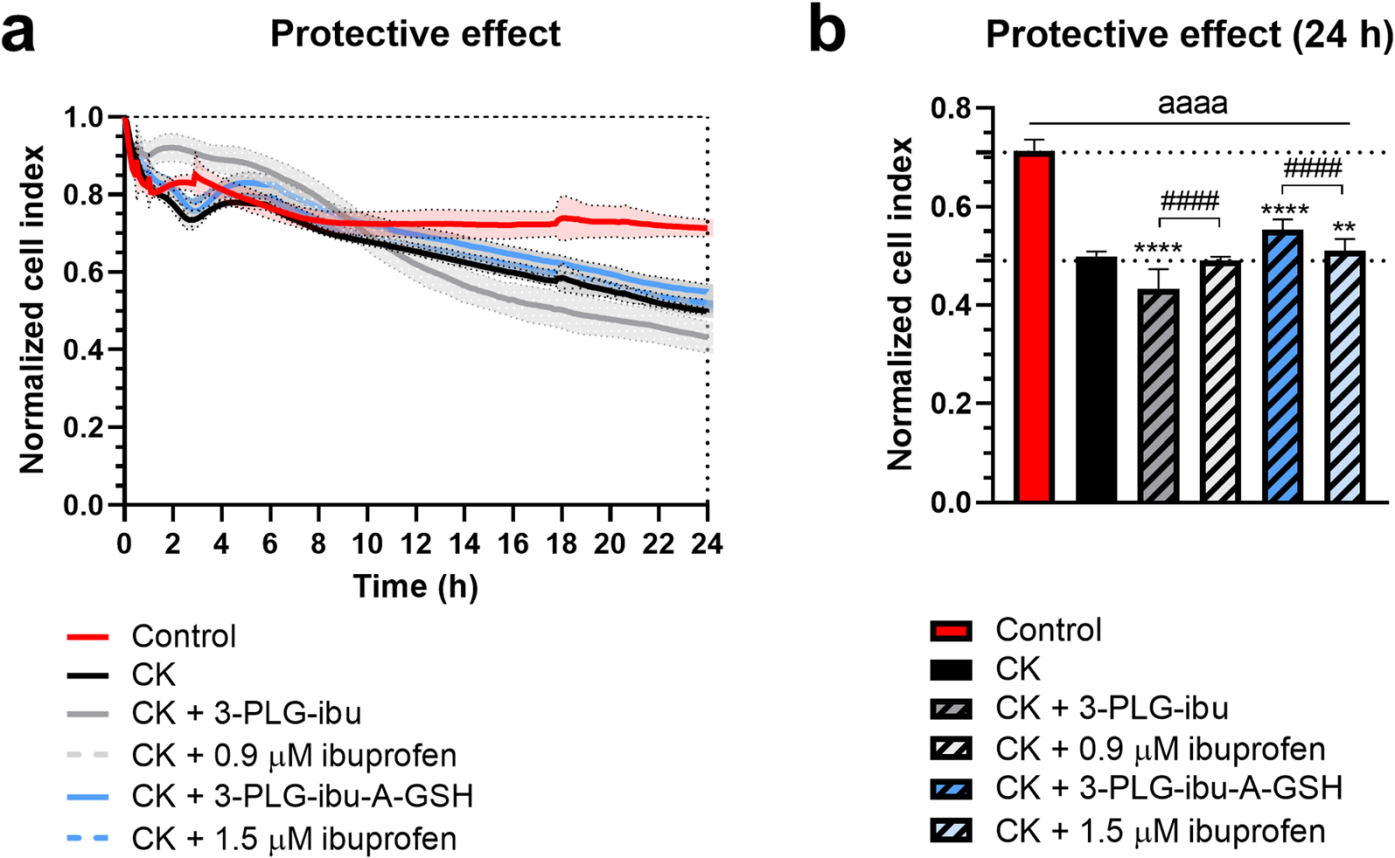
Protective effects of ibuprofen-coupled nanocarriers (3-PLG-ibu and 3-PLG-ibu-A-GSH) or ibuprofen against cytokine-induced (CK: TNF-α + IL1-β) barrier dysfunction on human brain endothelial cells. **a** Cell response kinetics monitored by real-time impedance measurements for 24 h. **b** Impedance of human brain endothelial cells at the 24-h time point. Values presented are means ± SD and are given as normalized impedance. Statistical analysis: one-way ANOVA followed by Tukey’s post-test; ^aaaa^ p < 0.0001 compared to the control group; **p < 0.01, ****p < 0.0001, compared to the CK group; ^####^p < 0.0001 between the nanocarrier and ibuprofen groups; n = 6–8

The changes in the barrier integrity of human brain endothelial cells after CK treatment alone and in co-treatments with ibuprofen or ibuprofen-coupled nanocarriers were followed by real-time impedance measurements for 24 h (Fig. 10a). One-day incubation of the cells with CK decreased the cell index by 30% compared to the non-treated control group (Fig. 10a, b) indicating a damaging effect on the barrier function of the cell layers. The co-treatment of the cells with 3-PLG-ibu (100 µg/ml) or 0.9 µM ibuprofen (the estimated drug content of 3-PLG-ibu nanocarrier) did not change the toxicity of CK treatment after 24 h. The targeted 3-PLG-ibu-A-GSH and ibuprofen at higher, 1.5 µM concentration (the estimated drug content of the 3-PLG-ibu-A-GSH nanocarriers) significantly protected against the barrier damaging effect of cytokines with the ibuprofen-coupled targeted nanocarrier group showing the highest effect against BBB dysfunction (Fig. 10a, b).

**Fig. 11:**
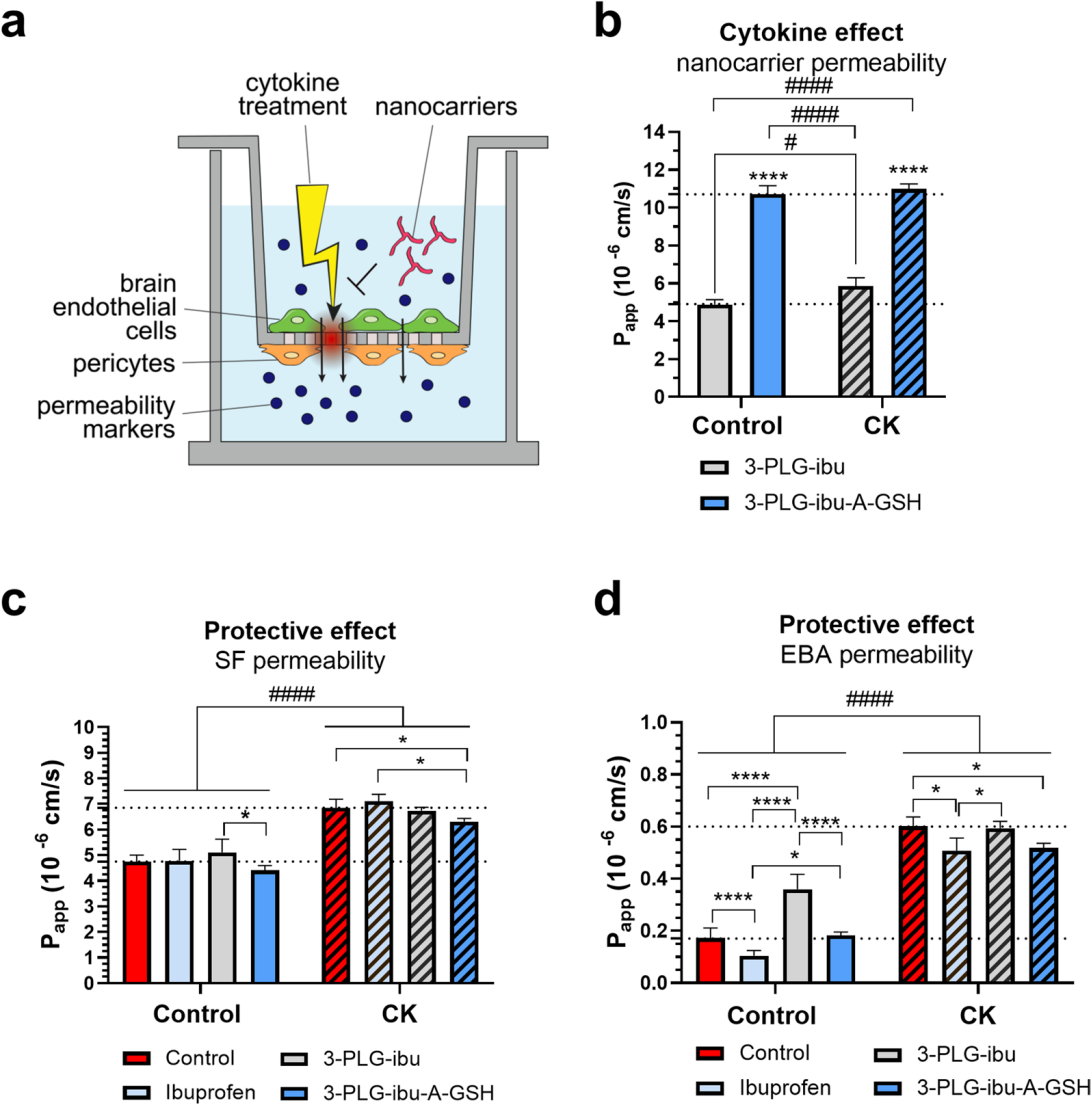
Protective effects of ibuprofen-coupled nanocarriers (3-PLG-ibu and 3-PLG-ibu-A-GSH) and ibuprofen against cytokine-induced (CK) barrier dysfunction in a human BBB co-culture model. **a** schematic drawing of the set-up. **b** Effect of CK on the permeability of 3-PLG-ibu and 3-PLG-ibu-A-GSH nanocarriers across the BBB model. Values presented are means ± SD. Statistical analysis: two-way ANOVA followed by Tukey’s post-test; comparisons within the treatment groups ****p < 0.0001; comparisons between the control and the CK groups; ^#^p < 0.05; ^####^p < 0.0001; n = 4. P_app_: apparent permeability coefficient. Penetration of **c** sodium fluorescein (SF) and **d** Evans blue-albumin (EBA) reference marker molecules after a 24-h permeability assay with nanocarriers or free ibuprofen (1.5 µM) with or without CK-treatment. Values presented are means ± SD. Statistical analysis: two-way ANOVA followed by Tukey’s post-test; comparisons within the treatment groups *p < 0.05; ****p < 0.0001; comparisons between the control and the CK groups; ^####^p < 0.0001 n = 4. P_app_: apparent permeability coefficient

The BBB permeability of ibuprofen-coupled nanocarriers was also tested under pro-inflammatory conditions (Fig. 11a). CK-treatment caused significant increase on the permeability of the non-targeted nanocarrier 3-PLG-ibu (5.86 × 10^–6^ cm/s vs. 4.89 × 10^–6^ cm/s in the control group), but it had no influence on the penetration of the targeted 3-PLG-ibu-A-GSH (10.99 × 10^–6^ cm/s vs. 10.72 × 10^–6^ cm/s in the control group; Fig. 11b). The targeted nanocarriers showed significantly higher permeability compared to the non-targeted nanoparticles both in control conditions and in the presence of cytokines.

The integrity of the BBB model was tested by permeability assays for SF and albumin marker molecules. The permeability of the small hydrophilic marker dye fluorescein was at the level of non-treated control (4.75 × 10^–6^ cm/s) in all groups at basal conditions which was significantly increased by 40% after cytokine treatment (Fig. 11c). This increased SF penetration was not reduced in the free-ibuprofen and the non-targeted nanocarrier groups, only the 3-PLG-ibu-A-GSH treatment decreased the elevated permeability in a statistically significant way (Fig. 11c).

CK-treatment increased 3.5-fold the permeability of albumin across the BBB model The penetration of the large biomolecule albumin showed similar pattern to the SF results with only two important significant differences. Firstly, the permeability of EBA increased after 24-h incubation with 3-PLG-ibu, and secondly, in inflammatory condition besides the 3-PLG-ibu-A-GSH the free ibuprofen treated group also significantly strengthened the integrity of the BBB model for the bigger transcellular marker molecule (Fig. 11d).

## Discussion

Our concept, which we verified in several previous paper [24, 32, 33, 42], is that if we mimic the specific expression pattern of nutrient transporters at the BBB with a combination of two or three different ligands a more specific targeting of nanoparticles across the BBB can be achieved. We confirmed both in vitro and in vivo that specific combination of ligands of different transporters, especially alanine and glutathione, is more efficient than single ligands to facilitate the penetration of nanocarrierss across the BBB [24, 32, 33, 42].

### Polymeric nanosystems for brain targeting

Polymeric nanosystems like poly(l-glutamic acid) nanoparticles have favorable properties for potential biomedical applications [23]. Despite the high drug loading capacity, or relatively easy modification with various ligands, this type of nanocarriers has been rarely investigated at the targeted brain drug delivery research field [50]. In our previous study we described that the A-GSH dual-targeting elevated the BBB penetration of 3-PLGs [24]. Coupling with drugs, fluorescent tracers or various targeting molecules can significantly change the physico-chemical properties and the intracellular fate of the nanocarriers [28, 51]. In our present follow-up study, we synthetized non-targeted and A-GSH functionalized 3-PLG nanoformulations coupled with dopamine and ibuprofen as active agents. To easily visualize and quantify 3-PLGs, all nanoparticles were labelled with R6G fluorescent dye.

We used a human BBB co-culture model improved by cARLA treatment, as described in our paper [40], for the impedance, cellular uptake and permeability experiments. Intensive and continuous pericellular claudin-5 staining was observed in the model and the shape of the hECs also indicated a typical brain endothelial cell layer (Fig. 2b), in agreement with our previous characterization of this BBB model [40]. The BBB expresses a large number of SLC transporters, among them the members of the ASCT and SNAT alanine carrier families [27]. The gene expression of five alanine carriers was identified on our human BBB model (Fig. 2c), suggesting that it is suitable for testing alanine-coupled nanocarriers. Regarding GSH, numerous research work verified the effectivity of this tripeptide as BBB targeting ligand of various vesicular [52–54] and poly(lactic-*co*-glycolic acid) (PLGA) nanoparticles [55, 56] in cell culture models, animals and in human studies as well. We also demonstrated that the permeability of GSH-targeted nanoparticles was increased across the cARLA-improved human BBB co-culture model [40] improving the predictive value of the current model for BBB penetration.

The covalent coupling of A-GSH targeting molecules, dopamine or ibuprofen drugs to the 3-PLG backbone by EDC/NHS reaction resulted in nanocarriers with hydrodynamic diameter in the 300–500 nm range (Fig. 3b). The nanocarrier samples were kept in lyophilized form and after dissolving showed similar size characteristic after 6-month storage at 4 °C (Table S1b). The transmission electron microscopy images confirmed branched, filamentous structures of the 3-PLG formulations (Fig. 3c). The determination of hydrodynamic diameter and PDI is very difficult in the case of multi-armed polymers, because the non-spherical shape of nanocarriers is one of the limitations of dynamic light scattering measurement technique [57]. Due to the negative effects of the sample preparation steps, like treatment with ethanol and air drying, on the polypeptide nanostructures transmission electron microscopy was also not suitable for proper sizing [58]. Size does not determine alone the fate of nanocarriers, the same permeability values were measured for polystyrene nanoparticles with 100, 200 or 400 nm diameter across a microfluidic BBB model [59]. In the same way, dopamine-loaded albumin/PLGA polymeric nanoparticles in the 300–400 nm size range were effectively targeted to brain and restored motor function in a mice model of PD [60]. In a comparative study the size dependency of BBB uptake and permeability was investigated for nanoparticles from 50 to 500 nm of various composition (polystryrene, protein, lipid) on a human culture BBB model in which transferrin nanocarriers with 500 nm diameter showed the most successful BBB penetration [61]. Nanocarriers for brain targeting were designed not only in the 100–200 nm but also in the larger, 300–500 nm size ranges [62]. We assume that the flexibility of the polypeptide backbone and the targeting ligands could help the uptake and permeability of the 3-PLGs with similarly greater size at the BBB model.

### Cellular internalization of targeted nanocarriers

The 3-PLGs nanoformulations were well-applicable in cellular assays and presented biological effects. The conjugation of dopamine or ibuprofen to the non-targeted or A-GSH functionalized nanocarriers did not affect the impedance kinetics of brain endothelial cells (Figs. S5, S6). A-GSH targeting resulted in a time-dependent increase in the cellular internalization of 3-PLGs conjugated with active agents (Figs. 4and 5) similarly to the uptake of 3-PLG-A-GSH without cargo in our previous paper [24]. During uptake assay co-incubation with molecules that support or interfere the SLC transport mechanism offers an excellent tool to investigate the characteristics of this pathway [27]. In our study, the presence of free alanine and/or GSH ligands reduced the internalization of nanocarriers 3-PLG-dopa-A-GSH and 3-PLG-ibu-A-GSH (Fig. 4) indicating the active role of A-GSH ligands and their transporters at the BBB during the cellular entry of targeted nanocarriers. Similar effect was described in case of dopamine-coupled quantum rods with carbohydrate shell that targeted the glucose transporter type-1 (GLUT-1) carrier [63]. Galactose ligand at high concentration decreased the internalization of nanoparticles in GLUT-1 expressing lung epithelial and human nasopharyngeal epidermal carcinoma cell lines [63]. GSH as a targeting molecule was described to increase the adhesion force between living brain endothelial cell surface and GSH-modified nanostructures measured by optical tweezer method [64]. Based on these data A-GSH targeting may increase the initial binding step of nanocarrier cell entry that in turn would lead to increased endocytosis.

To reveal the further steps of cellular internalization, the uptake of dopamine and ibuprofen coupled nanoconjugates was investigated with the application of non-selective inhibitors of endocytosis CD and Cyto D [65] and selective inhibitor of cytochrome c oxidase in the mitochondria sodium azide. [66] The molecule inhibits the metabolism of cells, and exploitable for investigation of ATP dependent cellular activities, such as nanocarrier internalization [67]. These compounds reduced the uptake of drug-coupled PLGs (Fig. 6a, b), as we observed in previous experiments with 3-PLG-A-GSH [24], and with serum albumin cargo loaded A-GSH targeted vesicular nanoparticles [32, 33]. We can conclude that endocytosis and active cell processes take part in the internalization of targeted 3-PLGs. Due to the overlapping inhibitory profile of CD and Cyto D [65], caveolae, clathrin mediated or macropinocytotic pathways may participate in the 3-PLG uptake in brain endothelial cells.

The surface charge of both the nanoparticles and the endothelial glycocalyx can modify the cellular entry of nanostructures [31]. Making the surface of hECs more positive with enzymatic digestion or treatment with cationic lipid decreased the uptake of the negatively charged 3-PLGs (Fig. 6c–e). Only the functionalization with A-GSH ligands of 3-PLG-dopa or 3-PLG-ibu elevated significantly the cellular uptake indicating a lesser role in physicochemical properties like charge for these nanocarriers. These results also suggest that charge dependent adsorptive mediated transcytosis, which is prominent for cationic molecules and carriers, may not play a strong role in the internalization of drug-coupled 3-PLGs.

### Transfer of nanocarriers across the BBB and biological effects

The main goals for BBB-targeted nanocarriers are at least three-fold, namely increase solubility, enhance brain penetration to reach better therapeutic efficiency, and minimize peripheral side-effects. The hydrophilic dopamine and lipophilic ibuprofen were used as model cargos in our experiments which present different solubility, permeability and side-effect problems.

Dopamine, which acts as a neurotransmitter in the CNS is well known to not cross the BBB [68] due to lack of specific transporter and active metabolism in brain endothelial cells. Dopamine also has strong cardiovascular effects and can induce hypertension. In contrast to the lack of brain entry of dopamine in vivo [68], our results with the targeted dopamine-nanoformulation using the BBB model combined with midbrain organoids indicate that the nanoformulation may help dopamine to reach the brain tissue after BBB penetration.

Functionalization with A-GSH increased the BBB permeability of both dopamine and ibuprofen copolypeptides across the human in vitro BBB model (Fig. 7b, c). In the literature mainly the receptor-mediated pathways were investigated to increase the BBB penetration of dopamine nanoformulations. Targeting liposomes with transferrin elevated the BBB penetration of dopamine across a human BBB culture model [69]. Angiopep-2, the ligand of the low-density lipoprotein receptor-related protein enhanced the BBB transcytosis of star-shaped, multibranched poly(l-glutamic acid) nanoconjugates in animals [50]. The carriers contained as active agents bisdemethoxycurcumin or genistein, and showed neuroprotective effects in the APP/PS1 mouse model of AD [50]. The combined targeting of the BBB carriers of alanine and the transporter(s) of GSH could offer an alternative pathway to increase the brain delivery of branched copolypeptides with dopamine cargo. We verified that 3-PLG-dopa-A-GSH not only had a significantly higher penetration across the BBB model than the non-targeted 3-PLG-dopa, but it also entered into healthy and PD midbrain organoids (Figs. 8 and 9). We described similar results in case of 3-PLG-A-GSH without dopamine coupling in the same experimental set-up [24]. These observations suggest that coupling dopamine to 3-PLG nanostructures did not alter the effective BBB penetration of A-GSH targeted 3-PLGs or entry to brain organoids. It is a limitation of the study that the midbrain organoid sets were generated from iPSCs from only one healthy donor and one PD patient. In our experiments no difference was found between the healthy and PD organoids regarding nanocarrier uptake. We should note however, that unless several donors are tested for each sets of organoids it is difficult to differentiate if experimental changes are due to allotypic variations between the donors or the disease-state.

Ibuprofen is insoluble in water, thus organic solvents such as ethanol, DMSO or dimethyl-formamide are needed for its pharmaceutic formulations that makes its safe applicability a challenge [70]. In connection with its high lipophilicity causing solubility problems ibuprofen poorly penetrates across the BBB. First, its high plasma protein binding limits brain uptake by reducing the free amount of the drug in the circulation [16]. Second, in the systemic circulation at pH 7.4 ibuprofen becomes ionized, leading to a substantial decrease in its lipophilicity. Thus, ibuprofen is too hydrophilic to efficiently cross the BBB by passive diffusion. This is supported by the very low brain to plasma ratio of ibuprofen, 0.02, in rats [16]. Same results were obtained on culture models, the P_e_ values of ibuprofen were 0.28 and 0.53 × 10^–6^ cm/s across a porcine brain endothelial cell line and a rat primary brain endothelial based BBB models, respectively [71].

Due to the key role of BBB dysfunction in neuronal diseases, protection of brain endothelial cells became a therapeutic target in brain pathologies [11]. Our group has also identified several protective molecules against BBB damage induced by glycation and oxidative stress [72], inflammation [73] and excitotoxicity [74]. Proinflammatory cytokines TNF-α and IL1-β activate the canonical NF-κB pathway that induce the gene expression of cyclooxigenase-2, inducible nitric oxide synthase-3, inflammatory cytokines, and matrix metalloproteinases [75] These changes lead to the damage of TJs, decreased endothelial integrity and increased permeability at the BBB [73, 76], and to neuroinflammation and the development of chronic neurodegenerative diseases such as AD [10, 77]. The damaging effects of proinflammatory CK were described and characterized in rat [66] or cARLA treated human BBB models [40]. The CK-induced decrease in the integrity of the BBB model was attenuated significantly by 3-PLG-ibu-A-GSH in all three types of experiments (Figs. 10 and 11), in contrast to ibuprofen or 3-PLG-ibu nanoparticles. This effect might be explained that targeted nanocarriers help the internalization of ibuprofen and its anti-inflammatory action in brain endothelial cells. Our observations are further supported by a study in APPswe/PS1dE9 mice [78]. A pegylated PLGA nanosystem loaded with the NSAID dexibuprofen partially crossed the BBB in a culture model, and significantly decreased the inflammation and β-amyloid plaques in the hippocampal region of AD mice [78]. Importantly, after three-month treatments of animals, the nanocarriers caused significantly lower gastric damage score than the free drug. These observations indicate that nanoformulations of ibuprofen or other NSAIDs can increase effectivity within the brain and at the same time minimalize the adverse peripheral side-effects [79].

## Conclusion

Here we presented a BBB-targeted nanodrug delivery system coupled with dopamine and ibuprofen. The results further proved the concept that A-GSH functionalization elevate the BBB penetration of polymeric nanocarriers potentially leading to better brain delivery of medicines. These findings will contribute to the development of advanced targeted polypeptide nanocarrier platforms for potential therapeutic application in neuronal diseases.

## Supplementary Information

Supplementary Materila 1: Fig. S1 Comparison of permeability across BBB models on cell culture inserts with different pore size. Fig. S2 ^1^H NMR spectra of **a** 3-armed poly(γ-benzyl-l-glutamic acid) (3-PBLG), **b** 3-armed poly(l-glutamic acid (3-PLG), and **c** A-GSH-targeted 3-PLG (3-PLG-A-GSH). Fig. S3 ^1^H NMR spectra of **a** 3-PLG-dopa, **b** 3-PLG-ibu, **c** 3-PLG-dopa-A-GSH, and **d** 3-PLG-ibu-A-GSH. Fig. S4 The calibration curve of R6G solution. Table S1a Characteristics of nanocarriers. **b** Changes in the size of dissolved nanocarriers after 6-month storage at 4 °C. Fig. S5 Effect of dopamine-coupled non-targeted (3-PLG-dopa) and alanine-glutathione-targeted (3-PLG-dopa-A-GSH) nanocarriers on the viability of human brain endothelial cells. Fig. S6 Effect of ibuprofen-coupled non-targeted (3-PLG-ibu) and alanine-glutathione-targeted (3-PLG-ibu-A-GSH) nanocarriers on the viability of human brain endothelial cells. Fig. S7 Effect of targeting ligands L-alanine, reduced L-glutathione and their combination on the cell impedance of human brain endothelial cells. Fig. S8 Live imaging of dopamine- (3-PLG-dopa; 3-PLG-dopa-A-GSH) and ibuprofen-coupled (3-PLG-ibu; 3-PLG-ibu-A-GSH) nanocarriers (yellow) and Golgi apparatus (magenta) in brain endothelial cells. Fig. S9 Effect of endocytosis and metabolic inhibitors on the cell viability of human brain endothelial cells. Fig. S10 Claudin-5 immunostaining (green) of human brain endothelial cells in co-culture model after permeability experiments (37 °C; 24 h) for 3-PLG-dopa and 3-PLG-dopa-A-GSH nanocarriers. Table S2 Barrier integrity for marker molecules fluorescein (SF) and albumin (EBA) after 24-h nanocarrier permeability assay. Table S3 Mass balance (%) values of permeability experiments.

## Data Availability

No datasets were generated or analysed during the current study.

## Abbreviations

^1^H NMR: Proton nuclear magnetic resonance
3-PBLG: 3-Armed poly(γ-benzyl-l-glutamic acid)
3-PLG: 3-Armed poly(l-glutamic acid)
3-PLG-A-GSH: 3-Armed poly(l-glutamic acid) coupled with alanine and glutathione
3-PLG-dopa: 3-Armed poly(l-glutamic acid) coupled with dopamine
3-PLG-dopa-A-GSH: 3-Armed poly(l-glutamic acid) coupled with dopamine and alanine and glutathione targeting ligands
3-PLG-ibu: 3-Armed poly(l-glutamic acid) coupled with ibuprofen
3-PLG-ibu-A-GSH: 3-Armed poly(l-glutamic acid) coupled with ibuprofen and alanine and glutathione targeting ligands
A-GSH: Alanine and glutathione targeting ligands
AD: Alzheimer's disease
ASCT: Neutral amino acid transporter
BBB: Blood–brain barrier
BLG NCA: L-glutamic acid γ-benzyl ester *N*-carboxyanhydride
BSA: Bovine serum albumin
cARLA: 8-(4-Chlorophenylthio)adenosine 3′,5′-cyclic monophosphate sodium salt + Ro-20-1724 + LiCl + A83-01
CD: β-Cyclodextrin
CK: Cytokines
CNS: Central nervous system
CytoD: Cytochalasin D
D_2_O: Deuterium oxide
DCM: Dichloromethane
DIW: Deionized water
DMEM: Dulbecco’s modified Eagle medium
DW: Distilled water
EBA: Evans blue-albumin complex
EDC/NHS: *N*-Ethyl-*N*ʹ-(3-(dimethylamino)propyl)carbodiimide/*N*-hydroxysuccinimide
EG_2_-diamine: 2,2′-(Ethylenedioxy)bis(ethylamine)
FBS: Fetal bovine serum
GLUT-1: Glucose transporter type-1
hEC: Human stem cell-derived endothelial cells
IL1-β: Interleukin-1β
LAT-1: L-type amino acid transporter-1
LRPs: Low density lipoprotein receptor-related proteins
NA: Neuraminidase
NSAIDs: Non-steroidal anti-inflammatory drugs
P_app_: Apparent permeability coefficient
PBS: Phosphate buffered saline
PD: Parkinson’s disease
PDI: Polydispersity index
PC: Pericytes
PLGA: Poly(lactic-*co*-glycolic acid)
R6G: *N*-(2-Aminoethyl) rhodamine 6G-amide bis(trifluoroacetate)
SF: Sodium fluorescein
SLC: Solute carrier
SNAT: Small neutral amino acid transporters
THF: Tetrahydrofuran
TMA-DPH: 1-(4-Trimethylammoniumphenyl)-6-phenyl-1,3,5-hexatriene
TMSI: Trimethylsilyl iodide
TNF-α: Tumor necrosis factor-α
