# Supplementary material for: Alanine and glutathione targeting of dopamine- or ibuprofen-coupled polypeptide nanocarriers increases both crossing and protective effects on a blood–brain barrier model
Source: Fluids Barriers CNS. 2025 Feb 19;22:18. doi: 10.1186/s12987-025-00623-2 (PMC11837687; doi:10.1186/s12987-025-00623-2)
Supplement: Supplementary file 1 — Supplementary Materila 1: Fig. S1 Comparison of permeability across BBB models on cell culture inserts with different pore size. Fig. S2 1H NMR spectra of a 3-armed poly(γ-benzyl-l-glutamic acid) (3-PBLG), b 3-armed poly(l-glutamic acid (3-PLG), and c A-GSH-targeted 3-PLG (3-PLG-A-GSH). Fig. S3 1H NMR spectra of a 3-PLG-dopa, b 3-PLG-ibu, c 3-PLG-dopa-A-GSH, and d 3-PLG-ibu-A-GSH. Fig. S4 The calibration curve of R6G solution. Table S1a Characteristics of nanocarriers. b Changes in the size of dissolved nanocarriers after 6-month storage at 4 °C. Fig. S5 Effect of dopamine-coupled non-targeted (3-PLG-dopa) and alanine-glutathione-targeted (3-PLG-dopa-A-GSH) nanocarriers on the viability of human brain endothelial cells. Fig. S6 Effect of ibuprofen-coupled non-targeted (3-PLG-ibu) and alanine-glutathione-targeted (3-PLG-ibu-A-GSH) nanocarriers on the viability of human brain endothelial cells. Fig. S7 Effect of targeting ligands L-alanine, reduced L-glutathione and their combination on the cell impedance of human brain endothelial cells. Fig. S8 Live imaging of dopamine- (3-PLG-dopa; 3-PLG-dopa-A-GSH) and ibuprofen-coupled (3-PLG-ibu; 3-PLG-ibu-A-GSH) nanocarriers (yellow) and Golgi apparatus (magenta) in brain endothelial cells. Fig. S9 Effect of endocytosis and metabolic inhibitors on the cell viability of human brain endothelial cells. Fig. S10 Claudin-5 immunostaining (green) of human brain endothelial cells in co-culture model after permeability experiments (37 °C; 24 h) for 3-PLG-dopa and 3-PLG-dopa-A-GSH nanocarriers. Table S2 Barrier integrity for marker molecules fluorescein (SF) and albumin (EBA) after 24-h nanocarrier permeability assay. Table S3 Mass balance (%) values of permeability experiments. [file 12987_2025_623_MOESM1_ESM.pdf]

Additional file 1

Alanine and glutathione targeting of dopamine- or ibuprofen-coupled polypeptide nanocarriers increases both crossing and protective effects on a blood-brain barrier model

Mária Mészáros[1,2], Thi Ha My Phan[3], Judit P. Vigh[1,4], Gergő Porkoláb[1,#], Anna Kocsis[1], Anikó Szecskó[1,4], Emese K. Páli[1], Nárcisz M. Cser[1], Tamás F. Polgár[1,5], Gábor Kecskeméti[6], Fruzsina R. Walter[1], Jens C. Schwamborn[7], Tamás Janáky[6], Jeng-Shiung Jan[3], Szilvia Veszelka[1*] and Mária A. Deli[1*]

Comparison of permeability across BBB models on cell culture inserts with different pore size

The pore size of cell culture inserts is an important parameter and we compared cell culture inserts with 3 µm and 0.4 µm pore sizes (Corning Costar; 0.33 cm^2) in a preliminary permeability test. As shown in Fig. S1 3-PLG-ibu-A-GSH nanocarriers penetrated across the BBB model on both insert types. Although the permeability values were lower in case of BBB models on the inserts with 0.4 µm pore size membranes, the significant difference (≈ 3.5) between the non-targeted and targeted groups remained the same. Moreover, there were no great differences between the penetration of trans- and paracellular markers across the BBB model on inserts with 3 µm pore-size membranes (EBA P_{app}: 0.17 x 10^{-6} cm/s; SF P_{app}: 3.22 x 10^{-6} cm/s) or 0.4 µm pore-size membranes (EBA P_{app}: 0.063 x 10^{-6} cm/s; SF P_{app}: 3.083 x 10^{-6} cm/s). Based on these results all further permeability assays were made on inserts with 0.4 µm pore size membranes.

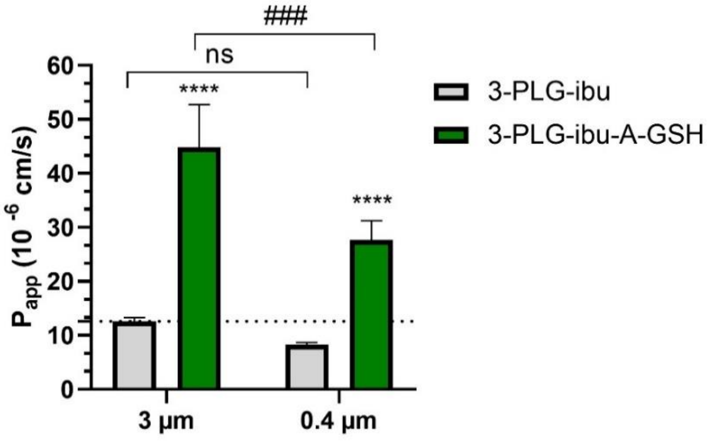

Fig. S1 Comparison of the permeability of ibuprofen-coupled nanocarriers across the human co-culture BBB model on inserts with 3 or 0.4 µm pore size membranes. Values are means ± SD. Statistical analysis: Two-way ANOVA followed by Tukey's test. **** $p < 0.0001$ compared to the 3-PLG-ibu; ### $p < 0.001$ between the 3-PLG-ibu-A-GSH groups; ns: non-significant. n = 4-5. P_{app}: apparent permeability coefficient.

Characterization of nanocarriers by proton nuclear magnetic resonance and ultraviolet-visible spectroscopy

As quality control, all products of nanocarrier synthesis were analyzed by proton nuclear magnetic resonance (^1H NMR) measurements (Fig. S2-3, Table S1a-b). Dopamine-coupled non-targeted and targeted 3-armed poly(L-glutamic acid) (3-PLG-dopa and 3-PLG-dopa-A-GSH) nanocarriers were dissolved in D_2O whilst the ibuprofen-coupled (3-PLG-ibu and 3-PLG-ibu-A-GSH) nanoformulations were dissolved in ethanol-d_6/D_2O (1:1) cosolvent mixture for ^1H NMR analysis. All nanocarriers were labeled with N-(2-aminoethyl) rhodamine 6G-amide bis(trifluoroacetate) (R6G) by using the EDC/NHS coupling reaction with a weight ratio of polypeptide to R6G at 20:1.

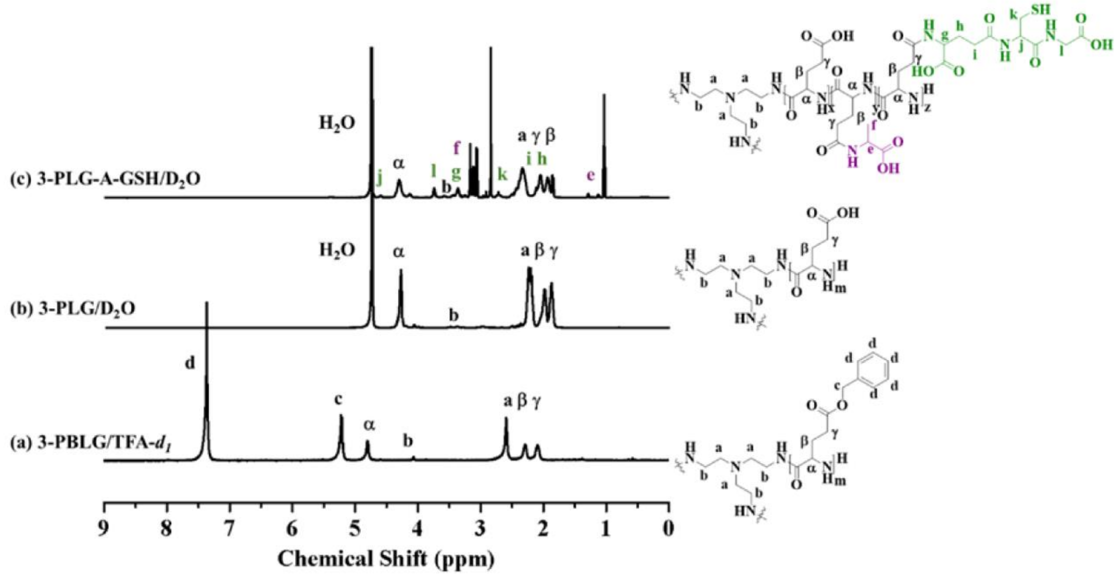

Fig. S2 ^1H NMR spectra of **a** 3-armed poly(γ-benzyl-L-glutamic acid) (3-PBLG), **b** 3-armed poly(L-glutamic acid (3-PLG), and **c** A-GSH-targeted 3-PLG (3-PLG-A-GSH).

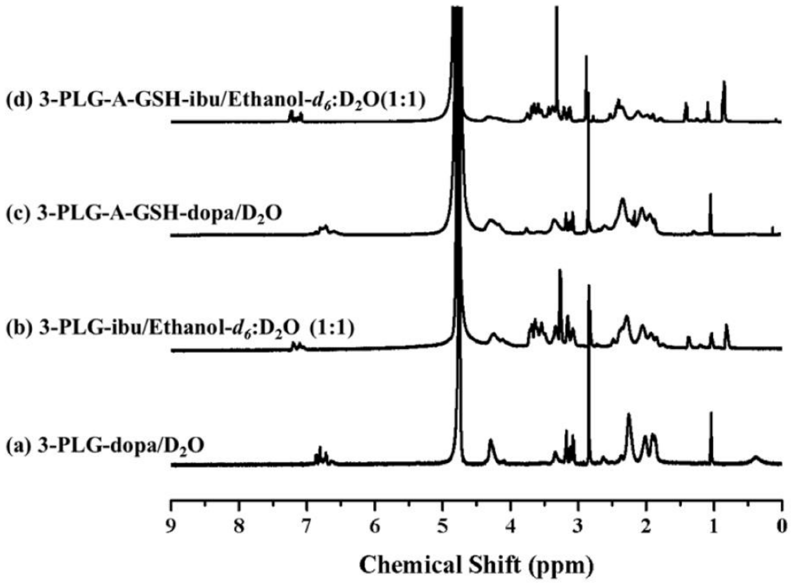

Fig. S3 ^{1}H NMR spectra of **a** 3-PLG-dopa, **b** 3-PLG-ibu, **c** 3-PLG-dopa-A-GSH, and **d** 3-PLG-ibu-A-GSH.

The concentration of grafted R6G in all copolypeptides was measured using UV-Vis analysis on a JASCO V-730 UV-Visible Spectrophotometer. R6G was prepared in ethanol/DIW cosolvent (1:1) at different concentrations (0.1, 0.5, 1.0, 2.0, and 5.0 ppm). The calibration curve was plotted using the absorbance values at the wavelength of 526 nm. The calibration curve of R6G solution at 526 nm is y = 0.1737x + 0.0018 (R^2 = 0.9998, y is absorbance, and x is the R6G content). The calibration curve was made based on UV-vis spectra of R6G at different concentrations (Fig. S4).

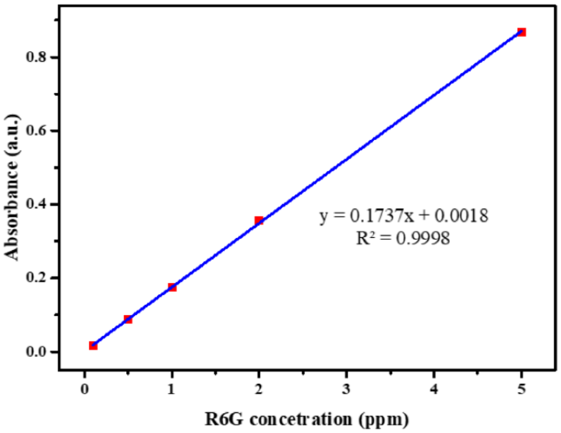

Fig. S4 The calibration curve of R6G solution. The absorbance of R6G at different concentrations in cosolvent of ethanol/DIW (1:1) was measured at the wavelength of 526 nm.

The concentrations of R6G in 3-PLG-dopa, 3-PLG-A-GSH-dopa, 3-PLG-ibu, and 3-PLG-A-GSH-ibu nanocarrier samples are shown in Table S1. The calculation of molecular mass and drug content values of nanocarriers were based on the degree of polymerization (DP) and grafting ratio of targeting ligands or drugs (Table S1a). The hydrodynamic diameter of samples was measured after 6-month storage at 4 °C by dynamic light scattering (Malvern Zetasizer Nano ZS, equipped with a He-Ne laser (λ = 632.8 nm) as described in the Materials and methods section of the main article (Table S1b).

Table S1a Characteristics of nanocarriers. Degree of polymerization (DP), grafting ratio of targeting ligands or drugs, concentration of R6G in samples were measured by proton nuclear magnetic resonance (^{1}H NMR) or ultraviolet-visible spectroscopy (UV-Vis).

**Samples**	**DP[a]**	**Grafting ratio[b]**				**Concentration of R6G[c] (%)**	**Molecular mass[d] (Da)**
		**A**	**GSH**	**dopa**	**ibu**		
**3-PLG-dopa**	**1:20.3**	-	-	0.28	-	0.313	8127
**3-PLG-dopa-A-GSH**		0.027	0.13	0.017	-	0.116	12052
**3-PLG-ibu**		-	-	-	0.07	0.012	8108
**3-PLG-ibu-A-GSH**		0.027	0.13	-	0.15	0.065	12086

[a,b] Calculated from ^{1}H NMR
[c] Calculated from UV-Vis data
[d] Statistical values

The observed decrease in the grafting ratio of dopamine in the 3-PLG-dopa-A-GSH sample compared to 3-PLG-dopa can be attributed to two factors. First, the modification of 3-PLG with alanine and GSH consumes free -COOH groups on the PLG backbone, which are required for the coupling of dopamine, thereby reducing its grafting efficiency. Second, the addition of alanine and GSH introduces steric hindrance around the backbone, further impeding the efficient coupling of dopamine. In contrast, the increase in the grafting ratio of ibuprofen in the 3-PLG-ibu-A-GSH sample compared to 3-PLG-ibu is likely due to improved solubility of the modified 3-PLG (grafted with alanine and GSH) in ethanol. The unmodified 3-PLG exhibits poor solubility in ethanol, which limits the coupling efficiency of ibuprofen. Upon modification, the enhanced solubility facilitates a more efficient reaction, resulting in a higher grafting ratio.

Table S1b Changes in the size of dissolved nanocarriers after 6-month storage at 4 °C.

**Nanocarriers**	**Size (nm)**		**PDI**	
	**freshly dissolved**	**6-month storage**	**freshly dissolved**	**6-month storage**
**3-PLG-dopa**	520 ± 109	534 ± 127	0.60 ± 0.11	0.49 ± 0.09
**3-PLG-dopa-A-GSH**	486 ± 104	344 ± 116	0.70 ± 0.09	0.62 ± 0.15
**3-PLG-ibu**	358 ± 100	354 ± 104	0.54 ± 0.11	0.83 ± 0.18
**3-PLG-ibu-A-GSH**	326 ± 64	523 ± 338	0.54 ± 0.15	0.65 ± 0.10

Minor changes in the size were measured after 6-month-storage compared to the freshly dissolved data of the non-targeted (3-PLG-dopa; 3-PLG-ibu) samples. The hydrodynamic diameter of 3-PLG-dopa-A-GSH showed decreasing, while the size of 3-PLG-ibu-A-GSH increased. The polydispersity index of dopamine-coupled nanocarriers decreased, the ibuprofen-functionalized polypeptides showed the opposite. We can conclude according to the size-changes of dissolved samples are relatively stable, usable after six months, but aggregation or damaging of the whole nanoparticle population could not be excluded.

Effect of nanocarriers, targeting ligands and endocytosis or metabolic inhibitors on human brain endothelial cells – impedance kinetics and MTT assay

The effect of nanocarriers, targeting ligands or cytochalasin D endocytosis inhibitor on the viability of stem cell-derived human brain endothelial cells was monitored by the measurement of impedance kinetics (RTCA-SP instrument, Agilent Technologies, USA) as described in the Materials and methods section of the main article. Confluent layers of brain endothelial cells at the plateau phase of growth were treated with 3-PLG-dopa, 3-PLG-dopa-A-GSH (10, 20, 100, 200, 1000 µg/ml) or 3-PLG-ibu, 3-PLG-ibu-A-GSH (1, 10, 20, 100 µg/ml) nanocarriers diluted in culture medium. To investigate the effect of targeting ligands, the cells were treated with 5 mM alanine or 5 mM GSH and their combination (A-GSH: 5+5 mM). The cells were incubated for 5 hours with cytochalasin D endocytosis inhibitor diluted in culture medium (0.3, 1, 3, 10 µg/ml).

MTT colorimetric cytotoxicity test [24] was used for investigation of randomly methylated β-cyclodextrin endocytosis and sodium azide metabolic inhibitors. Brain endothelial cells were cultured in 96-well plates (6×10^3 cells, Corning Costar) coated with collagen type IV and fibronectin in 50% pericyte-conditioned medium. At day 5 the confluent cell layers were supplemented with cARLA cocktail. Two days later the cells were incubated with randomly methylated β-cyclodextrin (1, 3, 5, 7.5 mM) or sodium azide (0.3, 1, 3, 10 mg/ml) diluted in culture medium at 37 °C for 5 hours in a CO_2 incubator. The MTT dye solution was prepared in phenol red-free medium at a 0.5 mg/mL final concentration. After the 5-hours incubation with inhibitors, the medium was removed and the MTT solution was added to the cells. The plates were incubated for 4 h at 37 °C. Formazan crystals, produced by living cells, were dissolved in 100 µl/well dimethyl sulfoxide on a horizontal shaker for 10 min. Dye absorbance was detected by a multi-well plate reader at a 570 nm wavelength (Fluostar Optima, BMG Labtechnologies, Germany). Cell viability was calculated as the percentage of dye reduction by culture medium-treated cells (control group).

The kinetics of cell responses to nanocarrier treatments are shown in Figure S5a-b. Except for the 3-PLG-dopa treatment at the highest 1000 µg/ml at 24 hours, we did not detect a decrease in the impedance of cell layers reflecting good cell viability and barrier properties (Fig. S5c). Normalized impedance values were not significantly reduced after 3-PLG-dopa-A-GSH incubation at any concentrations compared to the control group receiving culture medium at the 24-hour time-point (Fig. S5d).

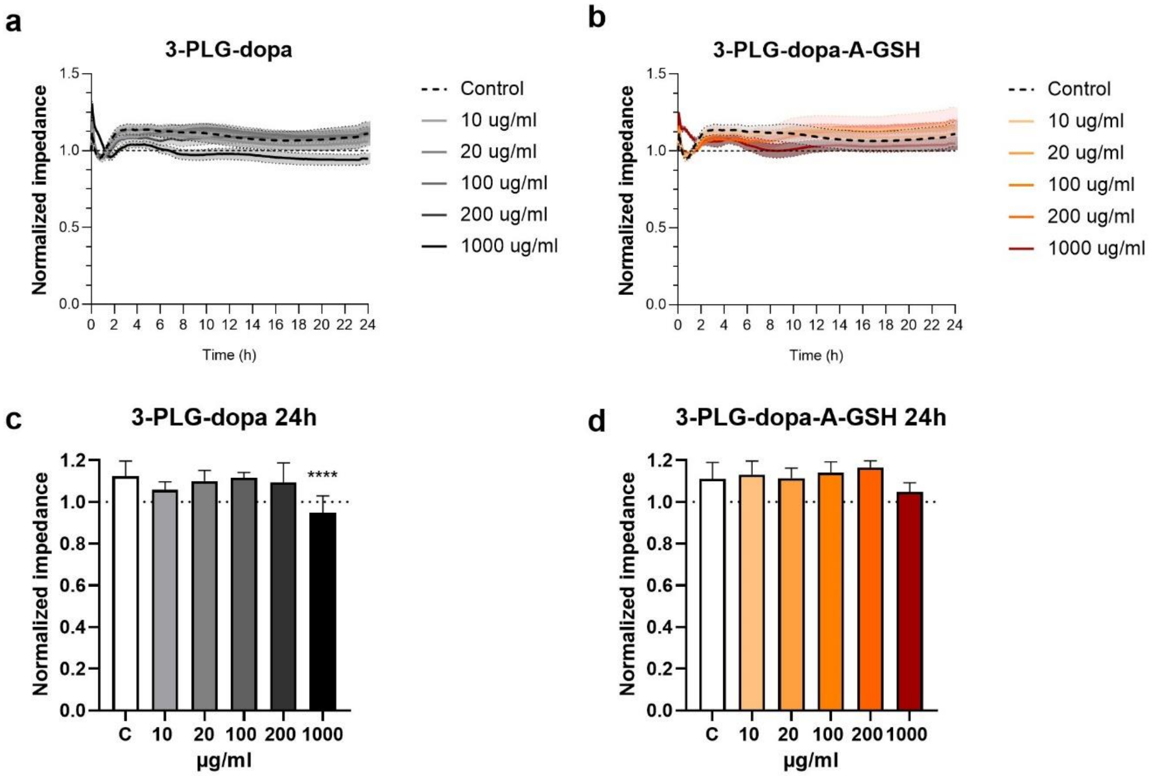

Fig. S5 Effect of dopamine-coupled non-targeted (3-PLG-dopa) and alanine-glutathione-targeted (3-PLG-dopa-A-GSH) nanocarriers on the viability of human brain endothelial cells. Impedance kinetics of cells treated with **a** 3-PLG-dopa and **b** 3-PLG-dopa-A-GSH in the concentration range of 10-1000 µg/ml monitored for 24 hours by real-time measurements. Impedance of human brain endothelial cells incubated with **c** 3-PLG-dopa and **d** 3-PLG-dopa-A-GSH at the 24-hour time point. Values presented are means ± SD and are given as normalized impedance. Statistical analysis: one-way ANOVA followed by Dunnett's post-test; **** $p < 0.0001$, compared to the control group; n = 6–8.

We monitored the response of human brain endothelial cells incubated with 3-PLG-ibu and 3-PLG-ibu-A-GSH in the concentration range of 1-100 µg/ml for 24 hours by real-time impedance measurements (Fig. S6a,b). The normalized impedance values of 3-PLG-ibu and 3-PLG-ibu-A-GSH groups at 24 h were not reduced as compared to the control group receiving culture medium (Fig. S6c,d), suggesting that nanocarriers did not exert a toxic effect on the cells. Moreover, at the 24-hour time-point the 3-PLG-ibu nanocarrier at 1, 20 and 100 µg/ml concentrations and the 3-PLG-ibu-A-GSH nanoparticle at100 µg/ml concentration significantly increased the impedance values of brain endothelial cells that may indicate a barrier tightening effect.

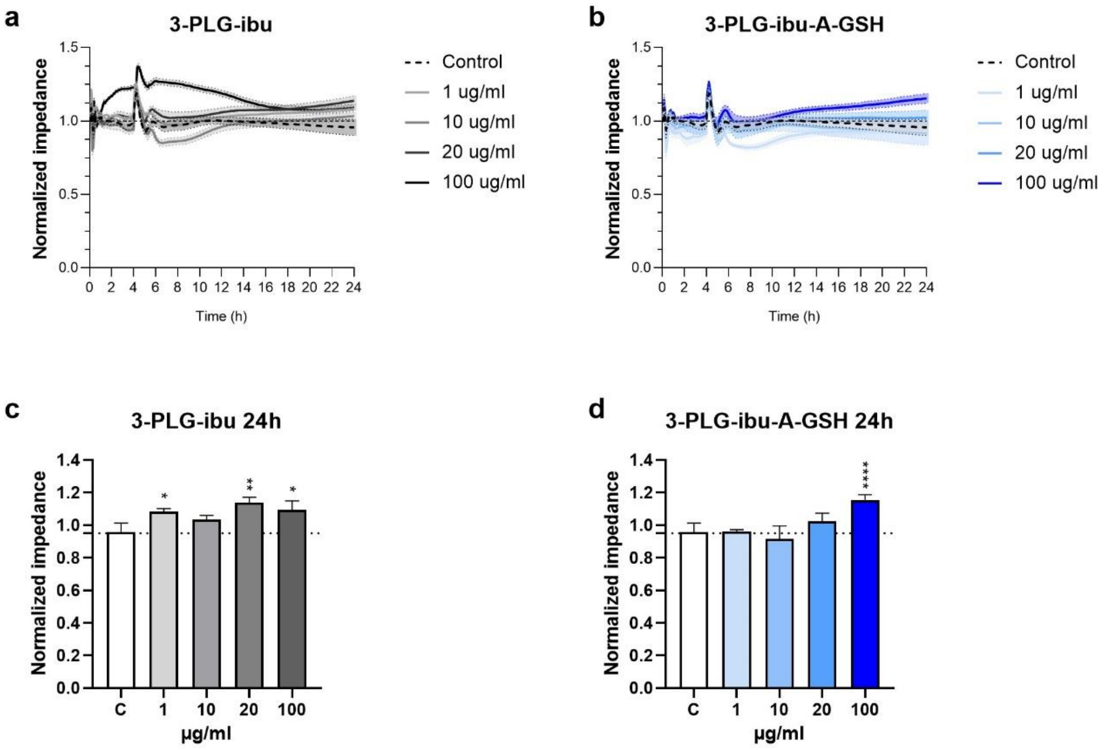

Fig. S6 Effect of ibuprofen-coupled non-targeted (3-PLG-ibu) and alanine-glutathione-targeted (3-PLG-ibu-A-GSH) nanocarriers on the viability of human brain endothelial cells. Impedance kinetics of cells treated with **a** 3-PLG-ibu and **b** 3-PLG-ibu-A-GSH in the concentration range of 1-100 µg/ml monitored for 24 hours by real-time measurements. Impedance of human brain endothelial cells incubated with **c** 3-PLG-ibu and **d** 3-PLG-ibu-A-GSH at the 24-h time point. Values presented are means ± SD and are given as normalized impedance. Statistical analysis: one-way ANOVA followed by Dunnett's post-test; * $p < 0.05$, ** $p < 0.01$, **** $p < 0.0001$, compared to the control group; n = 6–8.

The responses of human brain endothelial cell layers to targeting ligands alanine or GSH alone and their combination (A+GSH) were followed by real-time impedance measurements for 24 hours (Fig. S7a). Free ligands at high, 5 mM concentration neither alone nor in combination decreased the impedance after 4-, 8- and 24-hour incubation compared to the culture medium-treated control (Fig. S7b-d) suggesting no harmful or toxic effects on the cells. Interestingly, GSH and A+GSH incubation significantly increased the cell index at all time-points which may mirror the better barrier integrity of the human brain endothelial cells (Fig. S7b-d).

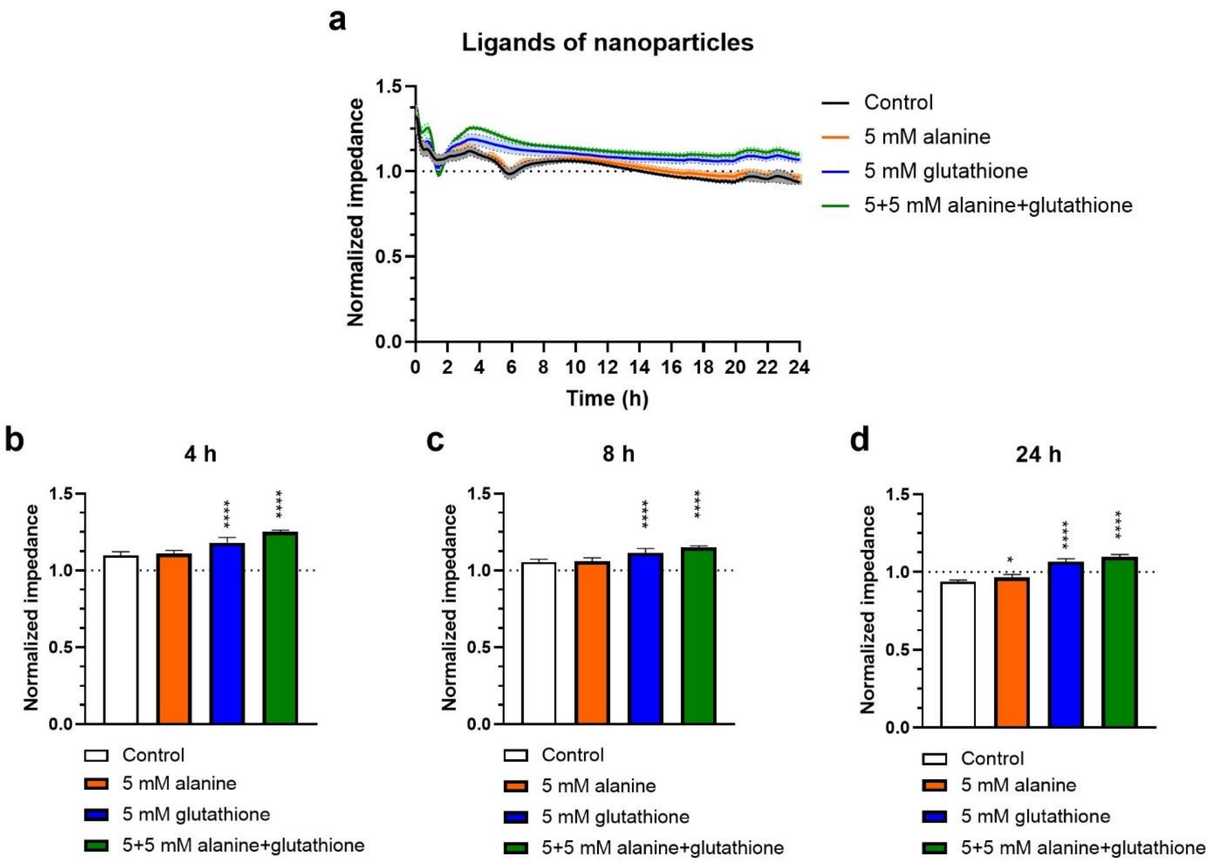

Fig. S7 Effect of targeting ligands L-alanine, reduced L-glutathione and their combination on the cell impedance of human brain endothelial cells. **a** Impedance kinetics of cell responses to ligands monitored for 24 hours by real-time measurements. Impedance of human brain endothelial cells incubated with L-alanine, reduced L-glutathione and their combination at the **b** 4-hour, **c** 8-hour, and **d** 24-hour time-points. Values presented are means ± SD and are given as normalized impedance. Statistical analysis: one-way ANOVA followed by Dunnett's post-test; * $p < 0.05$, **** $p < 0.0001$, compared to the control group; n = 6 - 8.

Colocalization assay of nanocarriers with Golgi apparatus

To investigate the intracellular trafficking of nanocarriers in living hECs, at the end of the 24-h uptake study the treatment solutions were supplemented with selective dye for Golgi apparatus (Golgi Staining Kit-Green Fluorescence-Cytopainter, Abcam, USA, ab139483, 1:100;

30 min) and Hoechst 33342 dye (1 µg/ml; 30 min) at 37°C in a CO_2 incubator. After the incubation, hECs were washed with Ringer-HEPES buffer (118 mM NaCl, 4.8 mM KCl, 2.5 mM $CaCl_2$, 1.2 mM $MgSO_4$, 5.5 mM D-glucose, 20 mM HEPES, pH 7.4) supplemented with 1% FBS. After washing step, the internalized non-targeted or targeted nanocarriers inside the living hECs were imaged using the 543 nm laser line on a Leica TCS SP5 confocal laser scanning microscope equipped with heated sample holder.

The image analysis was performed as previously described [24]. The colocalization of nanocarriers and Golgi was calculated by object-recognition based analysis with pixel-intensity correlation (object-corrected Pearson coefficient) following the protocol of Moser et al. [80].

The dopamine- (3-PLG-dopa; 3-PLG-dopa-A-GSH) or ibuprofen-coupled (3-PLG-ibu; 3-PLG-ibu-A-GSH) nanocarriers (yellow) and the Golgi apparatus (magenta) in living brain endothelial cells are shown in the representative pictures (Fig S8a). Nanocarriers, especially the targeted ones, can be seen in the cytoplasm of cells but not in the Golgi (Fig S8a). Based on image analysis, the co-localization area of Golgi and nanocarriers was limited (3-PLG-dopa: 6.7%; 3-PLG-dopa-A-GSH: 17.3%; 3-PLG-ibu: 10.5%; 3-PLG-ibu-A-GSH: 6.8%; Fig S8b).

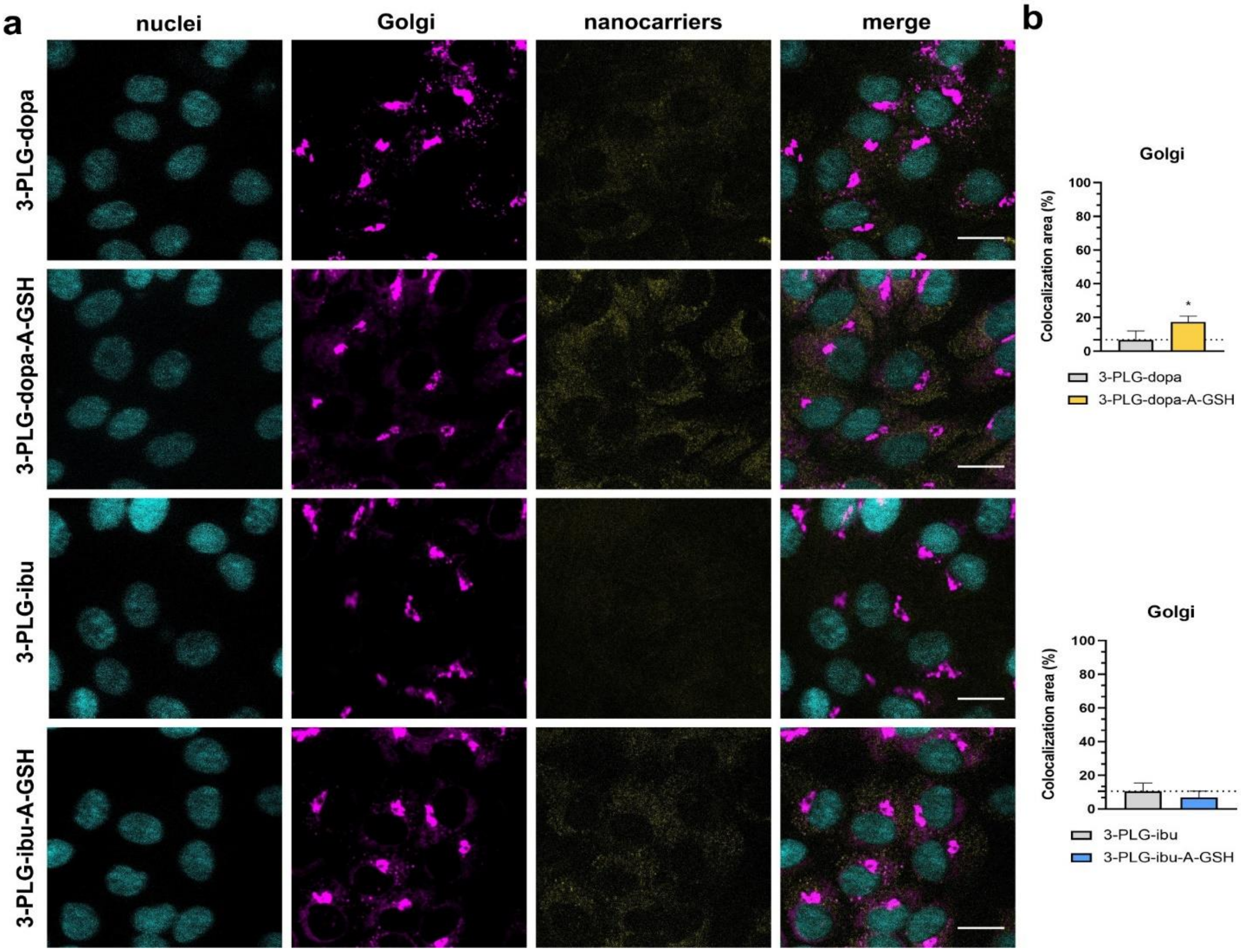

Fig. S8 Live imaging of dopamine- (3-PLG-dopa; 3-PLG-dopa-A-GSH) and ibuprofen-coupled (3-PLG-ibu; 3-PLG-ibu-A-GSH) nanocarriers (yellow) and Golgi apparatus (magenta) in brain endothelial cells. **a** Representative images of nanocarriers co-localized with Golgi apparatus. Cell nuclei are labeled with Hoechst 33342 (cyan). Scale bar: 20 µm. **b** Pixel-based image analysis of nanocarriers co-localized with

Golgi apparatus. Values are presented means ± SD and given as a percentage of co-localization area. Statistical analysis: unpaired t-test; *p < 0.05, compared to non-targeted groups; n=4-10.

The non-toxic treatment concentration of endocytosis and metabolic inhibitors for further experiments were determined by viability assays (Fig. S9a-c). At 5 hours, time length of uptake experiment with inhibitors and nanocarriers (Fig. 6.), ≤ 0.3 μg/ml cytochalasin D, ≤ 5mM randomly methylated β-cyclodextrin, and ≤ 3 mg/ml sodium azide are not toxic on cARLA matured human brain endothelial cells.

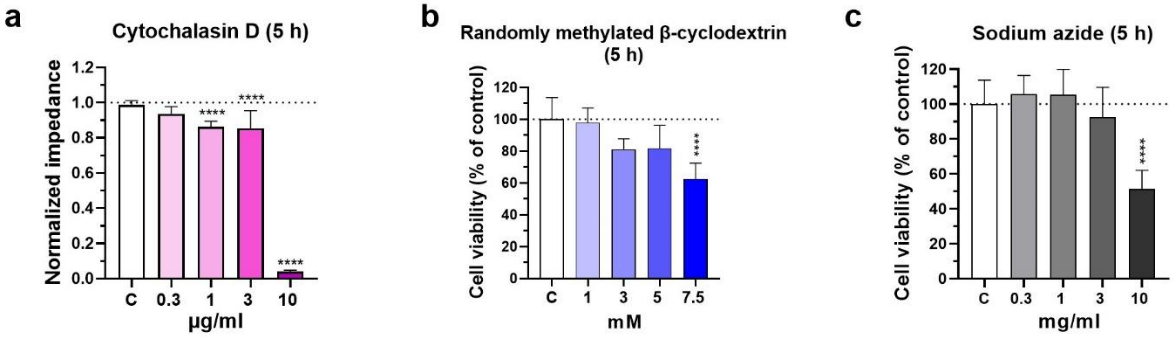

Fig. S9 Effect of endocytosis and metabolic inhibitors on the cell viability of human brain endothelial cells. **a** Normalized impedance of human brain endothelial cells incubated with Cytochalasin D endocytosis inhibitor at the 5-hour timepoint. Values presented are means ± SD and are given as normalized impedance values. Effect of cell viability after **b** randomly methylated β-cyclodextrin endocytosis or **c** sodium azide metabolic inhibitors on the cell viability at 5-hour timepoint measured by MTT test. Values presented are means ± SD and are given as a percentage of the control group. Statistical analysis: one-way ANOVA followed by Dunnett's post-test; **** p < 0.0001, compared to the control group; n = 6–8.

Barrier integrity after permeability assay with nanocarriers: immunostaining for claudin-5 protein

Immunocytochemistry of claudin-5 tight junction protein was performed on human brain endothelial cells after 24-h permeability assays with 3-PLG-dopa or 3-PLG-dopa-A-GSH nanocarriers (Fig. S10). The experimental set-up is shown in Figure 8a. At the end of the 24-hour permeability assays with 3-PLG-dopa and 3-PLG-dopa-A-GSH nanocarriers the cells on the culture inserts were fixed and immunostained as described in the Materials and methods section in the main article.

The claudin-5 junctional protein showed continuous pattern without any disruptions at the cell borders after permeability assays for both type of nanocarriers. The intact claudin-5 staining indicates that the barrier integrity of the human BBB model was well preserved during the assays and the nanocarriers did not damage the intercellular junctions.

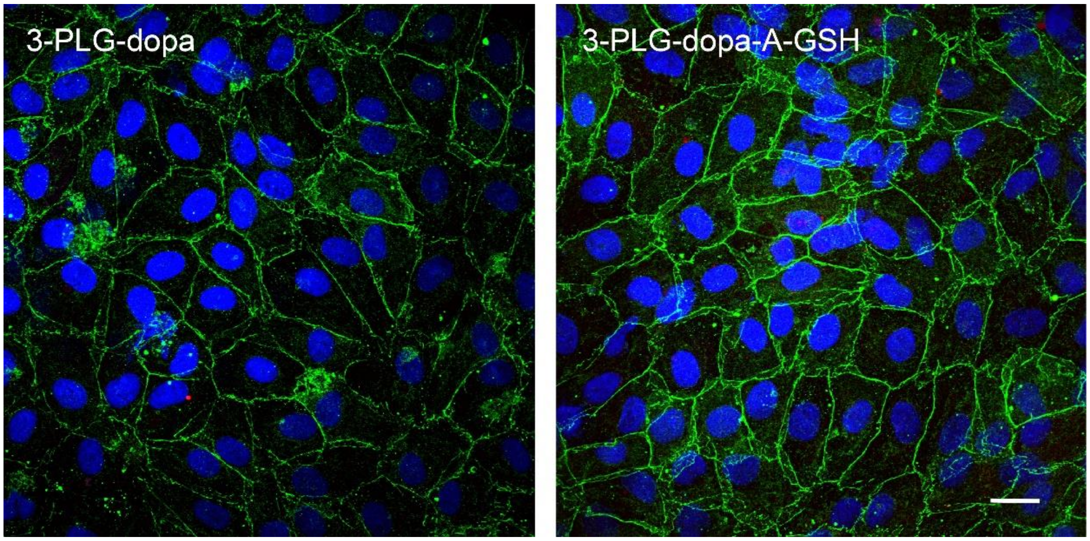

Fig. S10 Claudin-5 immunostaining (green) of human brain endothelial cells in co-culture model after permeability experiments (37 °C; 24 hours) for 3-PLG-dopa and 3-PLG-dopa-A-GSH nanocarriers. Cell nuclei are stained by Hoechst 33342 (blue). Scale bar: 20 µm.

Barrier integrity after permeability assay with nanocarriers: permeability of markers

In addition to the parallel permeability tests using a control group of the BBB model, a 30-min permeability test for fluorescein (SF) and albumin (EBA) markers was also performed on the BBB model after the 24-hour nanocarrier permeability assay presented in Fig. 7. According to the low levels of apparent permeability coefficient values (P_{app}) for paracellular SF and transcellular EBA, the integrity of the BBB model was preserved and the nanocarriers had no barrier damaging effect during the assay (Table S2).

Table S2 Barrier integrity for marker molecules fluorescein (SF) and albumin (EBA) after 24-h nanocarrier permeability assay.

**Nanocarriers**	**Average of apparent permeability coefficient (P_{app}; 10^{-6} cm/s)**	
	**SF**	**EBA**
**3-PLG-dopa**	1.82	0.07
**3-PLG-dopa-A-GSH**	1.75	0.13
**3-PLG-ibu**	2.89	0.14
**3-PLG-ibu-A-GSH**	1.39	0.03

Calculation of mass balance

The recovery values (mass balance) of samples in all nanocarrier permeability experiments were also calculated using the following equation:

$$\text{Mass balance (\%)} = \frac{C_{fA} V_A + C_{fB} V_b}{C_{0A} V_A} \times 100$$

where the C_{0A} is the initial concentration of the compound in the apical/donor compartment; C_{fA} is the final concentration of the compound in the apical/donor compartment; C_{fB} is the final concentration of the compound in the basal/acceptor compartment; V_A volume of the solution in the apical/donor compartment; V_b the volume of the solution in the basal/acceptor compartment.

High mass balance values were obtained for all nanocarrier groups, which indicates the good recovery of the nanoparticles from the permeability samples (Table S3) and no or low sequestration in the cells or binding to the plastic surfaces of the culture-ware. The lowest values were measured for 3-PLG-ibu samples, but it was still in the acceptable range for recovery.

Table S3 Mass balance (%) values of permeability experiments

**Experiments**	**3-PLG-dopa**	**3-PLG-dopa-A-GSH**	**3-PLG-ibu**	**3-PLG-ibu-A-GSH**
Permeability of nanocarriers (Fig. 7*)	103 %	103 %	55 %	82 %
Permeability of nanocarriers and entry into midbrain organoids (Fig. 8*)	82 % (WT organoids)<br>81 % (PD organoids)	93 % (WT organoids)<br>97 % (PD organoids)	-	-
Effect of cytokines on the permeability of nanocarriers (Fig. 11*)	-	-	61 % (no CK)<br>81 % (+CK)	91 % (no CK)<br>101 % (+CK)

*Figure numbers refer to the main article.

Quantification of ibuprofen by high throughput liquid chromatography-tandem mass spectrometry

Stock solutions of ibuprofen, ketoprofen (IS1), and naproxen (IS2) were prepared at a concentration of 1 mg/ml in methanol. Calibration standards were processed by spiking 400 µl permeability buffer (culture medium filtered through a 3 kDa Amicon Ultra centrifugal filter) with 20 µl diluted ibuprofen in the range of 0.01-2.5 µg/ml. In the same way 400 µL permeability buffer from the acceptor compartment was also filtered through a 3 kDa Amicon Ultra centrifugal filter. Standard points and samples were lyophilized, dissolved in 60 µl of 0.1% formic acid solution, spiked with 20-20 µl diluted IS1 and IS2 solutions (1 µg/ml) and extracted with 1 ml of ethyl

acetate-methyl-tert-butyl-ether (7:3) by vortexing for 10 min. After centrifugation the supernatant was moved to another microtube, and dried under N_2 gas at 40 °C. The residues were reconstituted in 50 µl of 50% methanol and analyzed by LC-MS/MS.

Liquid chromatographic separation was carried out on an Agilent 1100 Series nanoHPLC system (Agilent, Waldbronn, Germany) with an isocratic mobile phase consisting of 0.1% formic acid in water–acetonitrile (20:80, v/v) at a flow rate of 1.5 µl/min on a nanoEase Symmetry® C18 trap column (300µm × 25 mm, 5 µm; Waters Co., Milford, MA, USA). Mass spectrometric analysis was performed on a Q-Exactive™ Plus Hybrid Quadrupole-Orbitrap mass spectrometer (Thermo Fisher Scientific, Waltham, MA, USA) equipped with a nano-electrospray ion source. Mass spectrometer was operated with negative ionization and scheduled parallel reaction monitoring modes; transitions were monitored at m/z of 205.1 > 159.1 for ibuprofen, 253.1 > 208.9 for ketoprofen (IS1), and 229.1 > 169.1 for naproxen (IS2). Data acquisition and data processing were conducted using Xcalibur™ 4.1 (Thermo Fisher Scientific) and Skyline 24.1 softwares. The lower limit of quantification (LLOQ) was 50 ng/ml for ibuprofen with signal-to-noise (S/N) ratios > 10.

Quantification of dopamine by fluorimetric detection after liquid chromatographic separation of its o-phtalaldehyde derivatized product

Stock solution of dopamine was prepared at a concentration of 1 mg/ml in ethanol. Calibration standards were processed in the range of 0.5-10 µg/ml. Samples from the acceptor compartment (400 µL) in permeability buffer was concentrated by 3 kDa Amicon Ultra centrifugal filters. Standard points and samples were lyophilized, dissolved in 50 µl of 0.1% formic acid solution and analyzed by HPLC with fluorimetric detection after on-line pre-column derivatization with o-phtalaldehyde [81]. Liquid chromatographic analysis was carried out on an Agilent 1100 Series HPLC system (Agilent, Waldbronn, Germany) with gradient elution (30-45% B in 12 min) using mobile phases A: 0.1% formic acid in water and B: 0.1% formic acid in acetonitrile at a flow rate of 400 µl/min on a Kinetex PFP column (2.1 mm × 100 mm, 2.6 µm; Phenomenex, Torrance, CA, US) at 40 °C. Fluorescence emission was detected at 450 nm after excitation at 230 nm. The lower limit of quantification (LLOQ) was 0.5 µg/ml.

References

80. Moser B, Hochreiter B, Herbst R, Schmid JA. Fluorescence colocalization microscopy analysis can be improved by combining object-recognition with pixel-intensity-correlation. Biotechnol J. 2017;12(1):1600332. doi: 10.1002/biot.201600332.

81. Schuster R. Determination of amino acids in biological, pharmaceutical, plant and food samples by automated precolumn derivatization and high-performance liquid chromatography. J Chromatogr. 1988;431(2):271-84. doi: 10.1016/s0378-4347(00)83096-0.
